# Epigenetic dysregulation of enhancers in neurons is associated with Alzheimer’s disease pathology and cognitive symptoms

**DOI:** 10.1038/s41467-019-10101-7

**Published:** 2019-05-21

**Authors:** Peipei Li, Lee Marshall, Gabriel Oh, Jennifer L. Jakubowski, Daniel Groot, Yu He, Ting Wang, Arturas Petronis, Viviane Labrie

**Affiliations:** 1 Center for Neurodegenerative Science, Van Andel Research Institute, Grand Rapids, MI 49503 USA; 2 Centre for Addiction and Mental Health, Toronto, M5T 1R8 ON Canada; 3 Department of Genetics, Washington University in St. Louis, St. Louis, MO 63130 USA; 4 Institute of Biotechnology, Life Sciences Center, Vilnius University, LT-10257 Vilnius, Lithuania; 5 Division of Psychiatry and Behavioral Medicine, College of Human Medicine, Michigan State University, Grand Rapids, MI 49503 USA

**Keywords:** DNA methylation, Alzheimer's disease

## Abstract

Epigenetic control of enhancers alters neuronal functions and may be involved in Alzheimer’s disease (AD). Here, we identify enhancers in neurons contributing to AD by comprehensive fine-mapping of DNA methylation at enhancers, genome-wide. We examine 1.2 million CpG and CpH sites in enhancers in prefrontal cortex neurons of individuals with no/mild, moderate, and severe AD pathology (*n* = 101). We identify 1224 differentially methylated enhancer regions; most of which are hypomethylated at CpH sites in AD neurons. CpH methylation losses occur in normal aging neurons, but are accelerated in AD. Integration of epigenetic and transcriptomic data demonstrates a pro-apoptotic reactivation of the cell cycle in post-mitotic AD neurons. Furthermore, AD neurons have a large cluster of significantly hypomethylated enhancers in the *DSCAML1* gene that targets *BACE1*. Hypomethylation of these enhancers in AD is associated with an upregulation of *BACE1* transcripts and an increase in amyloid plaques, neurofibrillary tangles, and cognitive decline.

## Abstract (web summary)

Epigenetic control of enhancers may contribute to neurological disease. Here the authors carry out genome-wide analysis of DNA methylation in neurons isolated postmortem from patients with Alzheimer’s disease.

## Introduction

Alzheimer’s disease (AD) is an age-dependent chronic neurodegenerative disorder that is clinically characterized by the progressive deterioration of memory and cognitive functions. It is the leading cause of dementia, affecting 50 million people worldwide^1,2^. The primary neuropathological signs of AD are intraneuronal neurofibrillary tangles and extracellular β-amyloid (Aβ) plaques, along with accompanying synaptic and neuronal loss^2^. In general, the distribution of neurofibrillary tangles in the AD brain follows a stereotypic pattern; beginning in the entorhinal/perirhinal cortex, progressing to limbic structures including the hippocampus, and then finally spreading neocortically across the frontal, temporal, and parietal cortex^3^. Loss of neurons and severity of cognitive impairments in AD correspond closely with the burden of tangle pathology^4,5^. The neurodegenerative process is also mediated by excessive production and accumulation of Aβ peptides forming plaques. Generation of pathogenic Aβ peptides requires β-secretase (BACE1), which cleaves amyloid precursor protein (APP); the rate-limiting step in Aβ production^6^. Synaptic dysfunction in AD, which is evident long before substantial neuronal loss^7^, has been attributed to elevated BACE1 levels prompting the overproduction of toxic Aβ at synaptic terminals^6,8^. Recently, it has been demonstrated that Aβ plaques create an environment that enhances the aggregation of tau, which in turn forms intracellular neurofibrillary tangles^9–11^. Consequently, Aβ and neurofibrillary tangles jointly cooperate in the progression of AD^4,9,12^. However, AD is not a normal part of aging and the biological mechanisms causing some individuals, but not others, to develop disease pathology remain unclear.

The majority of AD is sporadic (>95%) in which aging is the strongest non-modifiable disease risk factor. Epigenetic mechanisms could contribute to AD, as many manifestations of aging, including age-dependent diseases, have an epigenetic basis^13,14^. Epigenetic marks like DNA methylation regulate gene transcription^13^, are responsive to environmental changes^15^, and show widespread remodeling during aging^16,17^. DNA methylation sites that robustly predict chronological age exhibit accelerated aging changes in the AD brain^17,18^, though these predictive sites have not been linked to specific effector genes. Moreover, in epigenome-wide studies, DNA methylation abnormalities identified in the AD brain are frequently located outside of gene promoters (and CpG islands)^14,19,20^. This begs the question of how do DNA methylation changes exert a pathogenic role in AD?

DNA methylation patterns controlling transcriptomic changes with age were recently found to prominently affect enhancer regulatory elements^21^. Enhancers are genomic elements that modulate the complex spatial and temporal expression of genes, and are subject to epigenetic regulation^13,22^. Enhancers, for the most part, are cis-acting and cell-type-specific, and often located outside of their target gene^13^. In the brain, epigenetically-controlled enhancers enable neuronal differentiation, experience-dependent gene transcription, and neuroplasticity^23–25^. Prior genome-wide studies examining DNA methylation changes in the AD brain report a significant overlap between differential methylation and enhancer elements^14,19^, suggesting that epigenetic disruption of enhancer function contributes to AD. However, the assay platform used in these prior studies primarily focused on CpG sites in coding regions (exons) and CpG islands^14,19,20,26^, and consequently, the majority of enhancer regions have yet to be examined in AD. In addition, CpG sites are not the sole location at which DNA methylation occurs. In human neurons, a large proportion of methylation occurs at non-CpG sites (CpH)^27^, yet the role of these non-canonical methylation sites in AD is unknown. Hence, in this study we perform a genome-wide analysis of DNA methylation, examining both CpG and CpH sites, at enhancers in neurons from AD brain. Our study, that specifically examines neurons, reveals novel gene regulatory regions involved in AD, and most importantly, provides insight into the mechanisms involved in AD pathogenesis.

## Results

### Differentially methylated enhancer regions in AD neurons

In order to identify enhancers involved in AD, we comprehensively mapped DNA methylation at enhancers, genome-wide, in neurons isolated from the prefrontal cortex of 101 individuals with no/mild, moderate and severe AD pathology (Braak stage: 1–2 *n* = 38 individuals, 3–4 *n* = 32, and 5–6 *n* = 31 individuals, respectively; Supplementary Table 1). We first isolated neuronal nuclei using an established antibody (NeuN+) and flow cytometry-based approach (Supplementary Fig. 1). We then fine-mapped DNA methylation at enhancer elements using a targeted bisulfite sequencing strategy, known as bisulfite padlock probe sequencing. For this, we designed 59,009 padlock probes covering all brain enhancers defined by the NIH Epigenomics Roadmap (active, poised/bivalent, weak, and genic enhancers; ChromHMM 18-state model). This padlock probe library also included brain promoters, since many act as enhancers^28^. In total, we examined 1,207,507 modified cytosines (122,071 CpGs and 1,085,436 CpHs) at 29,132 regulatory regions across the genome (Supplementary Fig. 2). We confirmed that % CpH methylation levels in our data were as previously reported^29^ and that there was not a selective neuronal subtype loss in the AD prefrontal cortex (Supplementary Figs. 2d, 3).

Our analyses focused on identifying differentially methylated enhancers associated with Braak stage, a standard measure of neurofibrillary tangle burden^3^. Braak stages 1–2 (transentorhinal), 3–4 (limbic), and 5–6 (neocortical) typically refers to unaffected individuals/clinically silent, incipient AD, and fully developed AD, respectively^3^. In our samples, clinical diagnosis of AD correlated closely with neurofibrillary tangle burden (Pearson’s correlation R = 0.78; *p* < 10^−15^), as expected^4,5^. In this analysis, we first identified differentially methylated cytosine sites (individual CpGs and CpHs) corresponding to the severity of tangle pathology (Braak stage), after controlling for age, sex, postmortem interval as well as neuronal subtype; the proportion of glutamate to GABAergic neurons in each sample, as determined by neuronal subtype deconvolution (Supplementary Fig. 3). There were 13 differentially methylated cytosines in AD neurons at *q* < 0.05 (robust linear regression model; Supplementary Table 2). We then parsed the targeted genome into regions (cytosines within 1000 bp; average region size: 261.5 ± 3.2 bp), and searched for regulatory regions with a significant enrichment of top 10% differentially methylated cytosines in AD neurons.

**Fig. 1:**
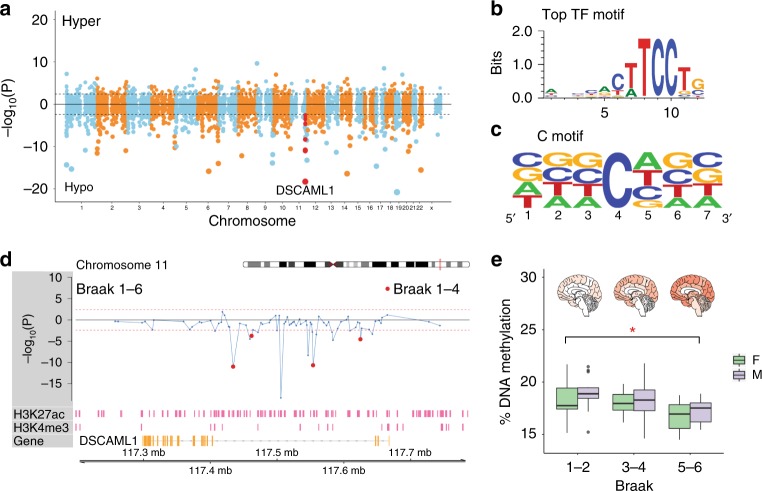
Fine-mapping of DNA methylation changes at enhancers, genome-wide, in AD neurons. In prefrontal cortex neurons of individuals with no/mild, moderate, and severe AD neurofibrillary tangle pathology (Braak stage 1–6; *n* = 101 individuals), DNA methylation was profiled at enhancer and promoter regions across the genome using bisulfite padlock probe sequencing. **a** Manhattan plot showing differentially methylated regions associated with the severity of neurofibrillary tangle pathology, after controlling for sex, age, postmortem interval, and neuronal subtype proportion. −log_10_(P) refers to the significance of differentially methylated cytosine enrichment at enhancers, with the sign corresponding to the direction of methylation change (hyper or hypomethylated). Threshold for genome-wide significance (dashed line) is *q* < 0.05. Enhancers in *DSCAML1* highlighted in red. **b** Image of ETS motif, the most strongly overrepresented TF motif within the differentially methylated enhancers in AD (*p* < 10^−18^, binomial distribution model in oPOSSUM). Motif sequence logo is provided by MotifMap^80^. **c** Contribution of CpG and CpH sites to DNA methylation differences at enhancers in AD. Motif analysis depicts a predominant contribution of CpH sites, especially CpA. **d** DNA methylation changes at enhancer regions in the *DSCAML1* gene in neurons that correspond to the severity of AD pathology. Dashed red line is genome-wide significance threshold (*q* < 0.05). Red dots denote enhancers that were also differentially methylated before the arrival of neurofibrillary tangles in the prefrontal cortex (Braak 1–4). Tracks for neuronal (NeuN + ) H3K27ac and H3K4me3, from PsychENCODE (pink, *n* = 9 individuals) are shown. **e** Averaged % DNA methylation across Braak stage for the most significantly disrupted enhancer region in *DSCAML1* (chr11: 117,504,514–117,506,898) in AD neurons. F = female; M = male. *q < 10^−14^, robust linear regression model followed by hypergeometric test. The boxplot center line is the median, the lower and upper limits are the first and third quartiles (25th and 75th percentiles), and the whiskers are 1.5× the interquartile range

There were 1224 regions showing differential methylation with increasing neurofibrillary tangle pathology (*q* < 0.05, robust linear regression model followed by hypergeometric test; Fig. 1a; Supplementary Data 1). Most epigenetically perturbed regulatory regions in AD neurons were hypomethylated (76.06%; 931 out of 1224 regions), and located far from any transcription start sites (TSS) in intergenic and exonic areas (average 50.5 ± 2.0 kb from a TSS; Supplementary Fig. 4), supporting a prevalent enhancer contribution to AD. Super-enhancers, which are clusters of enhancers that are magnitudes larger than common enhancers and help specify cell fate^30^, were not significantly overrepresented in the differentially methylated AD enhancers (*p* = 0.73, hypergeometric test). Transcription factor (TF) motif analysis showed that the ETS family, were most strongly overrepresented at the differentially methylated enhancers (*p* < 10^−18^, binomial distribution model in oPOSSUM; Fig. 1b); this TF family has a role in cell differentiation, cell cycle control, and apoptosis^31^.

We also examined the relative contribution of CpH sites to AD. In total, there were 5.8-fold more differentially methylated CpH sites (especially CpA) in AD enhancers than CpG sites (16,264 CpHs and 2803 CpGs; 1.49 and 2.30% of CpH and CpG sites tested, respectively; Fig. 1c). DNA methylation differences were on average larger at CpG sites (3.74% ± 0.05) than CpH (0.98% ± 0.01; *p* < 10^−15^, Welch’s *t*-test). Hence, CpH methylation is labile and together with CpG methylation is an important contributor to enhancer misregulation in AD neurons.

One of the most significantly disrupted regulatory elements in AD neurons was an enhancer located in intron 3 of the *DSCAML1* gene (chr11: 117,504,514–117,506,898; 1.56% hypomethylation in AD; *q* < 10^−14^, robust linear regression model followed by hypergeometric test; Fig. 1d, e). In fact, *DSCAML1* intron 3 contained the highest concentration of DNA methylation abnormalities in AD neurons affecting a total of 16 enhancers with cumulatively 304 significantly disrupted CpG/CpH sites in this 235.7 kb genomic area (0.62%–5.25% hypomethylation in AD; *q* < 0.05 to 10^−14^, robust linear regression model followed by hypergeometric test; Fig. 1d). These enhancer regions in *DSCAML1* intron 3 were all hypomethylated. This suggests that epigenetically activated enhancers in *DSCAML1* may have an important role in the progression of neurofibrillary tangle pathology and AD.

Next, we identified regulatory elements that were epigenetically altered prior to the arrival of neurofibrillary tangle pathology in neurons of the prefrontal cortex (Braak stage 1–4). There were 626 regulatory regions exhibiting significant DNA methylation changes (*q* < 0.05, robust linear regression model and hypergeometric test; Supplementary Data 1). Most notably, *DSCAML1* intron 3 contained 5 enhancers showing DNA hypomethylation occurring early in AD (0.70 ± 0.12% hypomethylation in AD; *q* < 0.05, robust linear regression model and hypergeometric test; Fig. 1d and Supplementary Fig. 5). Therefore, in prefrontal cortex neurons, hypomethylation of enhancers at *DSCAML1* precedes the onset of neurofibrillary tangle pathology.

### Gene targets of epigenetically misregulated enhancers in AD

**Fig. 2:**
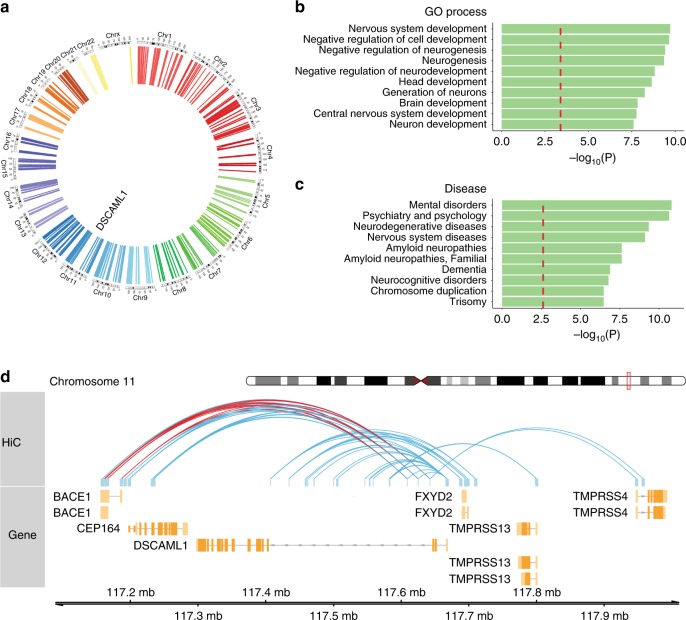
Chromatin conformation analysis in cortical neurons identifying the gene targets of the differentially methylated enhancers in AD. Chromatin interactions, genome-wide, were determined using Hi-C data from human prefrontal cortex. **a** Circos plot showing significant interactions between differentially methylated enhancers in AD neurons and gene promoters (TSS ± 2 kb). AD enhancers acted in cis for all their target gene promoters (average 587 kb ± 37 kb enhancer–target promoter distance; *n* = 1224 enhancers). **b**, **c** Pathways affected by genes with differentially methylated enhancers in AD, as determined by MetaCore. Top ten GO processes and disease pathways show that in our unbiased, genome-wide analysis, the enhancers dysregulated in AD neurons affected genes involved in neurogenesis and amyloid neuropathies (*q* < 0.05, hypergeometric distribution; red dashed line). **d** Chromatin interactions of the enhancers in *DSCAML1* that were differentially methylated in AD neurons (all interactions ± 400 kb of *DSCAML1* shown). Enhancers in *DSCAML1* targeted the promoters of *BACE1* (shown in red) as well as other genes

For enhancers to stimulate gene expression, the spatial configuration of the chromatin must bring enhancers in close proximity to their target gene promoters^22,32^. DNA methylation status at enhancers is an important determinant of enhancer**–**promoter interactions^32^, signifying that DNA methylation abnormalities at enhancers affect their ability to activate their cognate gene promoters. To uncover the gene targets of the epigenetically dysregulated elements in AD neurons, we investigated the 3D chromatin architecture in neurons. Using an in situ Hi-C dataset generated from human prefrontal cortex^33^, we found that our AD-relevant enhancer regions interacted with 1942 promoter regions (±2 kb from TSS), affecting 1207 genes (Fig. 2a; Supplementary Data 2). Enhancers disrupted in AD neurons were found to act in cis for all target promoters, with an average of 1.94 ± 0.11 promoters interacting with each enhancer. To maximize the identification of local interactions, we also used an in silico cis-regulatory region prediction tool to determine proximal genes associated with the AD enhancers. In total, 2431 genes were found to be potentially altered by aberrant DNA methylation at enhancers in AD neurons (Supplementary Data 1). Genes showing enhancer hypomethylation included the tau kinases: *MAPK10* (*JNK3*), *MARK3*, *CAMK2A*, *CAMK2B DYRK1A*, and the CDK5 neuronal activator *CDK5R2* (*p39*) and *CDK5RAP2*, which when overexpressed induces the hyperphosphorylation of tau that forms neurofibrillary tangles^34,35^.

We further examined the role of the genes with promoters targeted by the differentially methylated enhancers in AD neurons. Pathway analysis, using MetaCore, revealed a significant disruption of pathways involved in neurogenesis and neurodevelopment in AD (*q* < 0.05, hypergeometric distribution; Fig. 2b). In addition, amyloid neuropathies, dementia, and brain illnesses consisted of 8 of top 10 disease pathways (*q* < 0.05, hypergeometric distribution; Fig. 2c). Finally, genes with differentially methylated enhancers significantly converged with proteomic changes found in cortical neurons of AD patients captured by laser microdissection^36^. There were 109 genes with both an epigenetic disruption of their enhancer(s) and a protein change in AD cortical neurons (genes identified in the two datasets significantly overlapped, *p* < 10^−5^, hypergeometric test; Supplementary Fig. 6), indicative that there are functional consequences of aberrant DNA methylation at enhancers in AD neurons.

Given the extent of epigenetic disruption at enhancers in *DSCAML1* intron 3, we used the Hi-C dataset of prefrontal cortex to explore chromatin interactions in this genomic area. We found that enhancers in *DSCAML1* interacted with the *BACE1* gene promoter (Fig. 2d). Consequently, hypomethylation of these enhancers in neurons may underlie the overexpression of *BACE1* involved in AD pathophysiology^6^.

We also examined whether epigenetically misregulated enhancers in AD neurons were located near genetic polymorphisms (SNPs) identified in a large GWAS meta-analysis of AD. We first determined the linkage disequilibrium (LD) block (*R*^2^ > 0.8) for each of the 21 significantly associated AD SNPs (GWAS *p* < 10^−8^; Supplementary Table 3). None of the enhancers found differentially methylated in AD neurons directly overlapped these LD blocks. However, chromatin conformation analysis using the Hi-C dataset from prefrontal cortex demonstrated that three genes previously identified in GWAS had differentially methylated enhancers in AD neurons. These genes were: *BIN1*, *SORL1*, and *MEF2C* (Supplementary Table 3). Additionally, we ran a linear regression that compared the results of the entire AD GWAS (IGAP)^37^ and our DNA methylation study, adjusting for LD score. Our epigenomic study of enhancers in AD neurons had a significant positive correlation with AD GWAS (*p* < 0.01, linear regression). Thus, there are genes with both a genetic and epigenetic disruption in neurons that may contribute to AD.

### Transcriptional changes in genes with enhancer malfunction

We performed a transcriptomic analysis to further explore the effects of epigenetic alterations at enhancers in AD. RNA-sequencing (RNA-seq) was conducted on a subset of prefrontal cortex samples (*n* = 25 individuals) previously examined in our epigenetic investigation. Our analysis identified 1049 genes demonstrating differential expression with increasing neurofibrillary tangle pathology, after controlling for age, sex, postmortem interval, RNA quality (RIN), as well as neuronal variation (as determined by cell-type deconvolution; *q* < 0.05, robust linear regression model; Supplementary Data 3; Supplementary Fig. 7). We then determined whether enhancers exhibiting DNA methylation changes with AD pathology had corresponding changes in expression of their target genes. For this, we correlated enhancer DNA methylation change with increasing Braak stage to respective mRNA change with increasing Braak stage, adjusting for covariates (sex, age, postmortem interval, neuronal proportion, and for RNA-seq data RIN). We found that AD-associated enhancers exhibited DNA methylation changes with AD pathology that were inversely correlated to changes in target gene expression with AD pathology (*p* < 0.001, Pearson’s correlation; Supplementary Fig. 7c). We also examined whether differentially methylated enhancer regions in AD had a significant enrichment of differentially expressed enhancer RNAs (eRNAs) in AD. Using our RNA-seq data, we identified 2,563 brain enhancers with detectable eRNAs levels, of which 36 were differentially expressed in AD (*q* < 0.05, robust linear regression model; Supplementary Table 4). There was a significant enrichment of differentially expressed eRNAs at AD-associated enhancer regions (*p* < 0.05, hypergeometric test). This suggests that enhancers involved in AD exhibit corresponding differences in mRNA and eRNA levels with AD, though further analysis is required to fully explore eRNA changes in the AD brain.

**Fig. 3:**
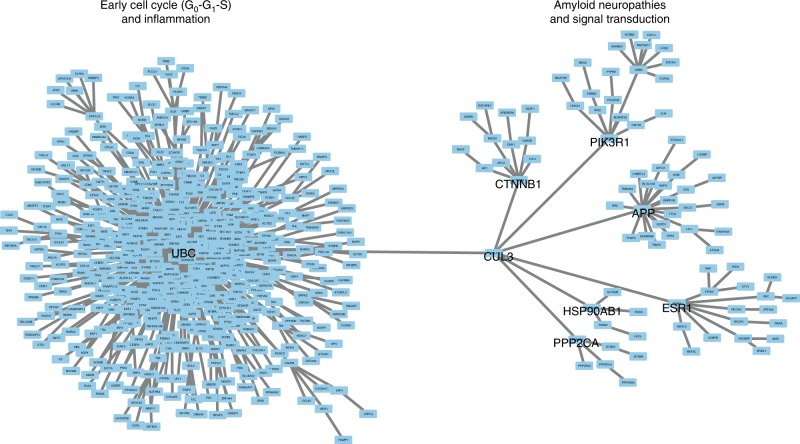
Omics analysis discovered high-confidence networks involved in AD. Using the FOREST OmicsIntegrator^77^, we merged our DNA methylation sequencing dataset profiling enhancers in AD neurons and transcriptomic sequencing data generated from the no/mild, moderate, and severe AD prefrontal cortex (Braak stage 1–6). Molecular pathways affected in AD neurons, as determined by network analysis are shown, and highlight two major networks involved in pathological progression in AD neurons. Pathway analysis by MetaCore of these two networks show their primary involvement in cell cycle reentry and amyloid neuropathies, respectively (*q* < 0.001, hypergeometric distribution). Hub genes: *UBC* and *CUL3;* subnetwork genes connected to *CUL3: APP*, *CTNNB1, PIK3R1, ESR1, HSP90AB1, and PPP2CA are* shown in black

Next, we integrated our epigenetic and transcriptomic dataset in a network analysis and found two major hubs converging on *UBC* and *CUL3*, which included *APP* as a subnetwork hub (Fig. 3; Supplementary Fig. 8). Pathway analysis of each hub showed an enrichment of 1) early cell cycle (G_0_–G_1_–S phase) and inflammation; and 2) amyloid neuropathies and signal transduction (Fig. 3; Supplementary Tables 5, 6; *q* < 0.05, hypergeometric distribution).

To validate our findings we examined a large RNA-seq dataset of the prefrontal cortex of AD patients and controls (*n* = 338 individuals) generated by the Religious Orders Study and Memory and Aging Project (ROSMAP)^38^. In this dataset, we found 1478 genes with differential expression corresponding to neurofibrillary tangle burden, after correcting for age, sex, postmortem interval, years of education, RIN, and neuron proportion (*q* < 0.05, robust linear regression model). There were 102 genes exhibiting significant transcriptional differences in the AD prefrontal cortex in both the ROSMAP and our dataset (differentially expressed genes in the two datasets significantly overlapped; *p* < 0.05, hypergeometric test; Supplementary Table 7), supporting the involvement of these genes in AD.

**Fig. 4:**
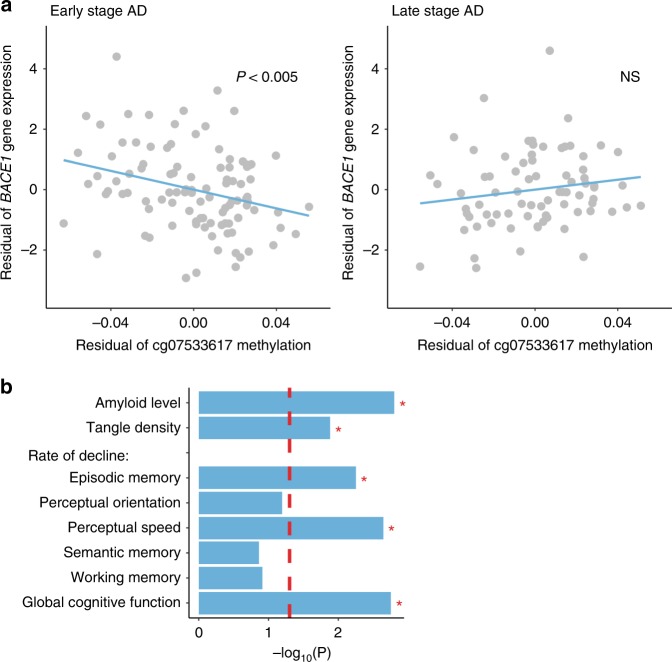
DNA methylation at *DSCAML1* intron 3 is linked to *BACE1* gene expression and to the pathology and cognitive symptoms of AD. **a** Correlation of DNA methylation at a *DSCAML1* CpG site (cg07533617) with *BACE1* mRNA expression in the AD prefrontal cortex. DNA methylation status at cg07533617 (situated in *DSCAML1* intron 3 at an enhancer interacting with the *BACE1* gene promoter) was inversely correlated with *BACE1* mRNA expression during the early stages of AD (Braak stage ≤ 4, *n* = 101 AD patients; *p* < 0.005, robust linear regression) but not in late stage (Braak stage ≥ 5, *n* = 76 AD patients; *p* = 0.21, robust linear regression). Analysis performed using the ROSMAP dataset, which contained both transcriptomic data generated by RNA-seq and genome-wide DNA methylation data generated by 450K Illumina DNA methylation arrays from the same AD patients. Correlation between residuals of DNA methylation (beta values of probe cg07533617, *x*-axis) and *BACE1* mRNA levels (FPKM, *y*-axis) shown. **b** Significance of correlation between DNA methylation at cg07533617 in *DSCAML1* intron 3 and AD pathology/cognitive symptoms; determined using ROSMAP DNA methylation and pathological/clinical data (*n* = 465 AD and controls; 251 AD and 214 controls). DNA hypomethylation at cg07533617 in *DSCAML1* intron 3 was significantly correlated with increased amyloid pathology and neurofibrillary tangle density, as well as a decline in episodic memory, perceptual speed, and global cognitive function (*p < 0.05, linear mixed model with annual cognitive measures as the longitudinal outcomes and DNA methylation as the predictor). **a**, **b** All analyses of ROSMAP data adjusted for sex, age, postmortem interval, years of education, neuronal cell proportion, as well as in **a** RIN

The ROSMAP^38^ also performed a DNA methylation analysis in the AD prefrontal cortex using 450K Illumina arrays. Though array coverage is sparse in *DSCAML1* intron 3 (22 CpG sites), there was one CpG (cg07533617) located at an enhancer found to target *BACE1* in our Hi-C analysis. Loss of DNA methylation at this *DSCAML1* CpG site was significantly associated with elevated *BACE1* mRNA expression in early stage AD pathology (Braak stage ≤ 4; *p* < 0.005, robust linear regression model; Fig. 4a). *BACE1* mRNA levels were no longer associated with *DSCAML1* methylation in late stage AD pathology (Braak stage 5–6; Fig. 4a). Likewise, correlation analysis between our DNA methylation and RNA-seq data (*n* = 25 individuals) supports that DNA methylation in this *DSCAML1* enhancer targeting *BACE1* influences *BACE1* mRNA levels (*q* < 0.05, robust linear regression model; Supplementary Fig. 9; Supplementary Table 8).

Moreover, in the ROSMAP data, we found that hypomethylation of the enhancer CpG site (cg07533617) in the *DSCAML1* intron 3 corresponded to an increased in amyloid plaque load, neurofibrillary tangle density, and a worsening of cognitive symptoms (*p* < 0.05, robust linear regression model for neuropathology and linear mixed model for cognitive symptoms; Fig. 4b). In particular, loss of DNA methylation at the *DSCAML1* CpG site was associated with the rate of decline in episodic memory, perceptual speed, and global cognitive function (*p* < 0.05, linear mixed model). Likewise, decreased DNA methylation across *DSCAML1* intron 3 (average of 22 CpGs) correlated with elevated *BACE1* mRNA expression in early AD (*p* < 0.001, robust linear regression model) and a rapid progression of cognitive symptoms (decline in perceptual speed; *p* < 0.05, linear mixed model; Supplementary Fig. 10). Therefore, an independent dataset supports that *DSCAML1* hypomethylation in AD neurons is associated with increased *BACE1* expression, which contributes to the development of AD pathology and symptoms.

### Genetic evidence for DSCAML1 enhancer regulation of BACE1

**Fig. 5:**
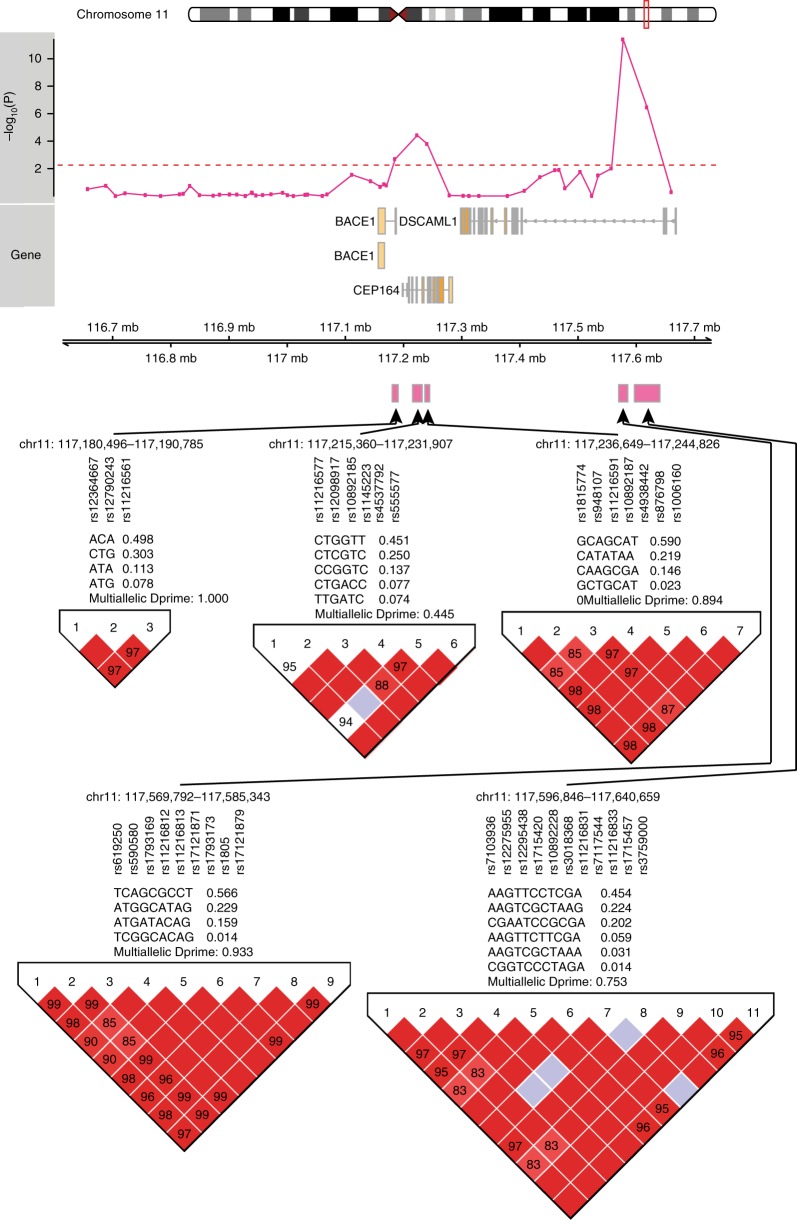
Genetic variation within *DSCAML1* intron 3 enhancers affects *BACE1* mRNA levels in the prefrontal cortex. ROSMAP data containing AD patients and controls (*n* = 278 individuals) that had both genotype data (using genome-wide SNP arrays) and transcriptome analysis (by RNA-seq). The extended genomic area around *BACE1* (±500 kb) is shown. Haplotypes (*n* = 53) determined by Haploview. Analysis of haplotype association with *BACE1* expression that controlled for age, sex, postmortem interval, Braak stage, years of education, RIN, and neuronal cell proportion was performed. Pink track shows the significance of the haplotype association with *BACE1* expression (dashed red line is *q* < 0.05). *BACE1* expression in the prefrontal cortex was influenced by two haplotypes in *DSCAML1* (*q* < 10^−4^, robust linear regression model). These two haplotypes overlapped four enhancers determined to be epigenetically dysregulated in AD neurons, supporting their involvement in *BACE1* regulation. Three haplotypes near the *BACE1* TSS were also associated with *BACE1* expression. Haplotype information (including SNP ID) and their population frequencies are shown for the five haplotypes significantly associated with *BACE1* expression. D’ values, measure of linkage disequilibrium, are shown in the boxes, with red boxes indicative of a strong linkage disequilibrium between SNPs

To further determine whether enhancers in *DSCAML1* impact *BACE1* expression in the brain, we examined the consequences of cis-acting genetic variation on *BACE1* gene expression (±500 kb area centered on *BACE1* and encompassing *DSCAML1*). For this, we used SNP information (*n* = 5533 SNPs determined by genome-wide arrays and imputation) and *BACE1* mRNA levels in the prefrontal cortex (determined by RNA-seq) of controls and AD patients from the ROSMAP study (*n* = 278 individuals). Our analysis identified 53 haplotypes in the extended *BACE1* genomic area, and examined their relationship to *BACE1* expression, after controlling for sex, age, postmortem interval, years of education, Braak stage, RIN, and neuronal proportion (Supplementary Data 4). We found two haplotypes within *DSCAML1* intron 3 (chr11: 117,569,792–117,585,343; chr11: 117,596,846–117,640,659) influencing variability in *BACE1* expression (*q* < 10^−4^, robust linear regression model; Fig. 5; Supplementary Fig. 11), as well as another three haplotypes near the *BACE1* TSS (*q* < 0.05, robust linear regression model; chr11: 117,180,496–117,190,785; chr11: 117,215,360–117,231,907; chr11: 117,236,649–117,244,826). The two haplotypes in *DSCAML1* intron 3 linked to *BACE1* expression overlapped 4 enhancers that were hypomethylated in AD neurons. This supports that enhancers in *DSCAML1* are directly involved in *BACE1* regulation, and thereby may contribute to the development of AD pathology and symptoms.

### Epigenetic changes at enhancers contribute to aging

Advanced age is universally considered to be the most salient risk factor for AD^2^. Why aging is the strongest risk factor for AD is not well understood, particularly at a mechanistic level. Previous whole-genome bisulfite sequencing (WGBS) analysis of DNA methylation in healthy prefrontal cortex neurons demonstrated that CpH methylation, but not CpG methylation, dramatically rises in early life, coinciding with the period of synaptogenesis and brain maturation^27^. We therefore investigated whether CpH methylation sites in AD enhancers exhibits age-dependent changes in later life to promote the development of AD.

**Fig. 6:**
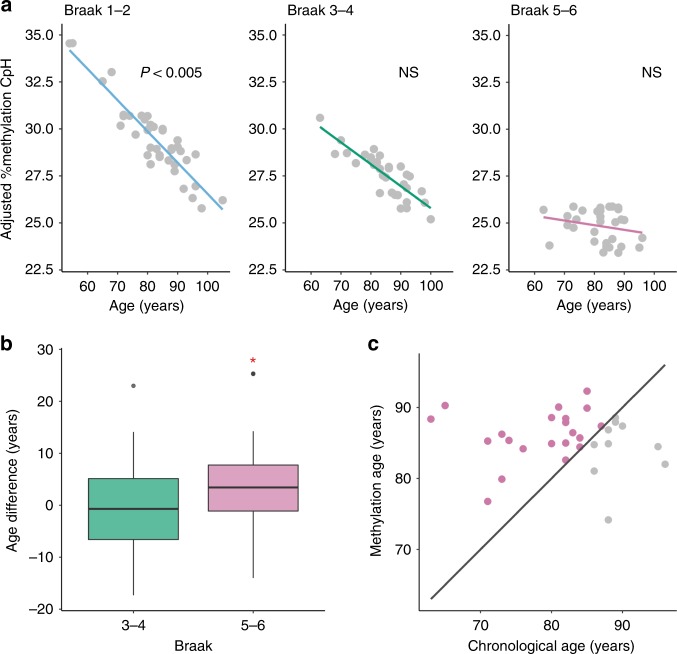
Accelerated CpH methylation changes with aging in AD neurons. Age-dependent changes in CpH methylation in neurons from individuals with no/mild, moderate, and severe AD pathology (Braak stage 1–2, 3–4, 5–6, and *n* = 38 individuals, 32, and 31 individuals, respectively). Age analysis was performed on CpH sites (*n* = 3661 CpHs) in enhancers relevant to AD (*n* = 848 enhancers showing both epigenetic and associated gene transcript differences in AD, as identified by Omics integration analysis). **a** Aging changes in % CpH methylation at enhancers in neurons. CpH methylation decreased with age in individuals with no/mild AD pathology (*p* < 0.005, robust linear regression model), but not in moderate or severe AD. Analysis of % CpH methylation changes with age adjusted for sex, postmortem interval, and neuronal proportion. **b** Box plot showing the difference between the epigenetic age and chronological age in neurons of moderate and severe AD cases. The epigenetic aging calculator^17^ used CpH sites to determine the epigenetic ages of the moderate and severe AD groups. CpH methylation aging was accelerated in severe AD neurons (*p* < 0.05, paired *t*-test). The boxplot center line is the median, the lower and upper limits are the first and third quartiles (25th and 75th percentiles), and the whiskers are 1.5× the interquartile range. **c** CpH methylation age relative to chronological age in neurons of severe AD cases (Braak stage 5–6). Each dot represents a severe AD case, and the line demarks where chronological age matches epigenetic age. Purple dots are samples in which epigenetic age is greater than chronological age. Severe AD cases typically show an epigenetic age of 80 years or older regardless of chronological age

First, we confirmed that there is an increase in CpH methylation in AD-associated enhancers in neurons during early life. Using a WGBS dataset of healthy human prefrontal cortex neurons^27^, we found that between infancy and adulthood there was a 2.01-fold increase in CpH methylation at AD enhancers in neurons, which then remained stable during middle age, as previously reported^27^ (Supplementary Fig. 12). We next examined the aging dynamics of CpH in our cohort of older adults (54–105-years-old), focusing on 3,661 CpH methylation sites across 848 enhancers found most relevant to AD in the epigenetic and transcriptomic integration analysis (Fig. 3). We found that in neurons of the prefrontal cortex, CpH methylation significantly changed with age, after controlling for sex, postmortem interval, Braak stage, and neuron subtype proportion (*p* < 10^−3^, robust linear regression model). However, epigenetic aging differed depending on severity of AD pathology. In neurons of healthy individuals, CpH methylation steadily declined with age (Braak stage 1–2; 1.50% hypomethylation; *p* < 0.005, robust linear regression model; Fig. 6a); an effect that was less evident in moderate AD (Braak 3–4, *p* = 0.16, robust linear regression model). However in neurons of individuals with advanced AD (Braak stage 5–6, *p* = 0.46, robust linear regression model) CpH methylation had already reached a low level and no longer changed with age in older adults (Fig. 6a).

We then asked whether the lack of decline in CpH methylation in severe AD neurons was due to an acceleration in epigenetic aging. To test this, we used the epigenetic clock, which uses DNA methylation patterns to evaluate divergences between biological and chronological aging^17^. With this model, we used our enhancer CpH sites from healthy individuals (Braak stage 1–2) to predict the age of advanced AD cases (Braak stage 5–6). We found that neurons of advanced AD cases have a significant 3.67 ± 1.61 year acceleration in DNA methylation aging (81.87 ± 1.42 years for chronological age vs 85.54 ± 0.70 years for predicted biological age; *p* < 0.05, paired *t*-test; *n* = 31 individuals; Fig. 6b). Interestingly, though chronological age varied in advanced AD (range: 63–96 years-old), the neurons of nearly all these cases exhibited an epigenetic age exceeding 80-years-old (Fig. 6c). Therefore, healthy and AD neurons exhibit differential epigenetic aging. In healthy neurons, CpH methylation at enhancers increase in early life, and then subsequently decrease with advanced age. However, AD neurons experience an accelerated loss of CpH methylation at enhancers, and as a result are epigenetically old.

## Discussion

Our highly detailed deep-sequencing maps capturing both CpG and CpH sites at enhancer and promoter elements in neurons of AD patients and controls serves as a rich resource for exploring disrupted gene regulation in AD neurons. First, we demonstrated widespread epigenetic abnormalities at enhancers in AD neurons that largely involved the loss of CpH methylation, most frequently at CpA sites. CpH methylation is as effective as CpG methylation in repressing gene transcription and is depleted in active enhancers and promoters^39,40^, signifying that loss of CpH methylation at enhancers in AD typically represents an activation of target genes. Second, we find that genes with epigenetically disrupted enhancers in AD significantly converge on transcriptomic changes observed both in our sample cohort and those of a large, independent study (and also strongly overlaps proteomics abnormalities in AD neurons). Integration of our omic datasets supports that AD neurons exhibit abnormal cell cycle reentry, and have an overstimulation of apoptotic and inflammatory pathways. Third, we identified enhancers responsible for *BACE1* overexpression in AD neurons, and provided a molecular link between excessive Aβ production and the progression of neurofibrillary tangle pathology. Finally, we demonstrate that in aging neurons of older adults, there is a loss of CpH methylation at enhancers, which when accelerated may progress AD. Together, our study supports that epigenetic dysregulation of enhancers occurs early in AD neurons, and disrupts the activity of key genes involved in the pathological onset and progression of AD.

Enhancer hypomethylation in AD neurons prominently affected genes involved in neurogenesis pathways. In early life, CpH methylation, including CpA methylation, is gained in neurons during a period of major synaptic restructuring^27,41^. CpA methylation has been shown to preferentially accumulate at lowly transcribed genes, particularly those involved neurodevelopment and axon guidance, later recruiting MECP2 to restrain the transcription of these genes in adulthood^41^. This accumulation of CpA likely enables the developing brain to progress from a stage of widespread neuronal proliferation and migration to finely-tuning synapse formation and pruning. However, in post-mitotic, terminally-differentiated neurons, reactivation of cell cycle pathways induces apoptosis rather than proliferation^42^. Our data showed an evident epigenetic and transcriptional loss of cell cycle control in AD neurons. This includes upregulation of cyclin-dependent kinases (CDKs), which has previously been shown to promote neuronal death^43,44^. Abortive attempts of neurons to reenter the cell cycle via *CDK5* activation causes persistent synaptic loss, neurodegeneration, and AD-like cognitive deficits in mouse models^15,45^. In addition, upregulation of cell cycle genes mediates the hyperphosphorylation and aggregation of tau, which forms neurofibrillary tangles^46,47^. Therefore, loss of CpA and other methylated cytosines at enhancers prompts the reactivation of cell cycle and neurogenesis pathways, which leads to tauopathy and pro-apoptotic effects in post-mitotic neurons. Enhancer hypomethylation affecting neurogenesis genes may underlie the close association between neurofibrillary tangle burden and neuronal loss occurring in AD.

If ectopic cell cycle reentry in neurons induces tauopathy and neurodegeneration, then what facilitates the epigenetic reactivation these cell cycle genes? Evidence in mice models demonstrates that early intraneuronal accumulation of Aβ peptides promotes global DNA hypomethylation and the activation of cell cycle reentry (i.e., via CDK5)^48–50^. Similarly, differentiated human neurons exposed to Aβ show DNA methylation abnormalities that are most prevalent at cell-fate genes controlling neuronal differentiation/apoptosis^51^. Pathological and imaging studies support an early onset of Aβ plaques in AD, typically preceding the spread of neurofibrillary tangles and neurodegeneration^52,53^. In addition, the presence of Aβ plaques fuels the spread of tau pathology across the neocortex by amplifying the formation of tau species capable of seeding new aggregates^9,10^. This signifies that Aβ peptides not only form plaques, but may jointly promote the pathogenic loss of DNA methylation at enhancers involved in cell cycle reactivation, and stimulate the propagation of tau pathology in the brain. Driving the overproduction of Aβ peptides in the AD brain is BACE1. In this study, we found that AD neurons have extensive loss of DNA methylation at enhancers located in *DSCAML1* intron 3. Chromatin conformation analysis revealed that enhancers in *DSCAML1* targeted the *BACE1* gene promoter. Hypomethylation of these enhancers occurred early in AD, and corresponded to an aberrant upregulation of *BACE1* expression early on in disease. Hence, epigenetic activation of enhancers in *DSCAML1* is an early disease event that may lead to the overproduction of Aβ peptides, which in turn is capable of facilitating the improper reactivation of cell cycle genes and neuronal loss^48–51^. In support, DNA methylation changes within *DSCAML1* corresponded closely to the development of AD symptoms.

**Fig. 7:**
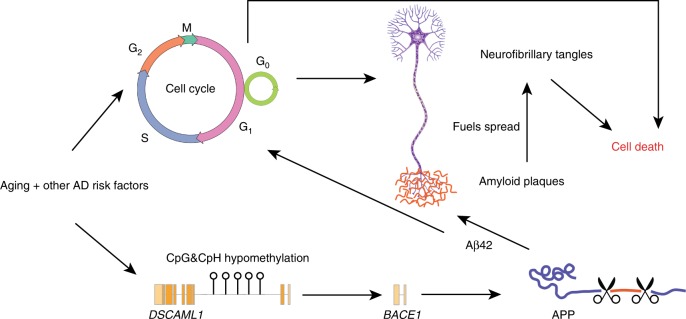
Proposed model displaying the contribution of enhancer dysregulation to the development and progression of AD. In aging, neurons gradually lose CpH methylation at enhancers, especially at enhancers regulating genes involved in the early cell cycle. Aging and other AD risk factors that trigger improper attempts of neurons to renter the cell cycle promotes the formation of neurofibrillary tangles in the AD brain and is neurotoxic. In AD neurons there is also a hypomethylation of enhancers in *DSCAML1* that occurs early in disease. Enhancers in *DSCAML1* activate *BACE1* expression, which leads to the increased production of the Aβ peptides. Aβ peptides promotes an accelerated loss of DNA methylation at enhancers in AD neurons, thereby facilitating aberrant cell cycle reactivation. Aβ peptides also forms plaques, which fuels the spread of neurofibrillary tangle pathology across the brain. Consequently, epigenetic changes at enhancers in AD neurons contributes to the pathology, neurodegeneration, and cognitive symptoms of AD

From our findings and the existing literature described above, we speculate the following model: hypomethylation of enhancers in *DSCAML1* activates *BACE1* to induce the formation and progression of both Aβ plaques and neurofibrillary tangle pathology in AD (Fig. 7). Interaction of *DSCAML1* enhancers with their target *BACE1* promoter leads to overproduction of Aβ peptides, which will eventually form plaques. Aβ peptides in turn engages the hypomethylation of enhancers affecting neurogenesis and cell cycle genes, which are already primed for activation due to the normative loss of CpH methylation marks with aging. Reactivation of cell cycle genes facilitates tau hyperphosphorylation, and along with Aβ plaques, leads to the formation and spread of tangle pathology, resulting in neuronal death and AD cognitive symptoms.

Notably, the DNA methylation changes at enhancers identified in this study represent molecular signatures of the pathogenic process occurring in AD neurons, though the epigenetic analysis does not delineate causal vs secondary effects. However, we did find 626 regions exhibiting significant DNA methylation changes at enhancers in AD neurons prior to the arrival of pathology, which support an early involvement of the epigenome in disease processes. This included enhancers at *DSCAML1*, which had an epigenetic status closely linked to the *BACE1* transcript levels specifically in early AD. A study limitation is that our study sample size of 101 individuals led to a limited number of individually significant methylated cytosines, and hence this study focused on enhancer regions with significant enrichment of top differentially methylated cytosines. However, both the individual cytosine and region analysis supported that the *DSCAML1* intron 3 enhancers were differentially methylated in AD neurons. Moreover, the integration of epigenetics and transcriptomic data, the replication of our findings with independent proteomics dataset of cortical neurons^36^, as well as further characterization with the large, independent ROSMAP data^38^ that included clinical AD symptoms outlines a useful approach for epigenomic studies of brain diseases. In addition, our deep-sequencing DNA methylation maps emphasize the dynamic regulation of enhancer elements in human brain neurons occurring with age and AD progression.

Our investigation of the molecular changes occurring in AD neurons has the potential to advance disease-modifying treatments for AD. Currently, there are ongoing clinical trials testing the suitability of BACE1 inhibitors for the treatment of prodromal AD, with the mindset that inhibiting BACE1 blocks one of the earliest pathogenic events in AD^54^. However, concerns regarding cognitive side effects and safety have been raised in regards to abolishing the healthy physiological functions of BACE1 and the potential spill-over onto BACE2^55^. An alternative AD treatment could be to inhibit excessive *BACE1* activation via the enhancers in *DSCAML1*, which would treat the pathological trigger of *BACE1* overexpression, and may consequently minimize side effects. Compounds involving enhancer-RNA downregulation or zinc finger protein-based approaches to block inappropriate enhancer activity are early in development^56,57^, but have been shown to cross the mammalian blood-brain-barrier, and similar therapies show promising effects in clinical trials for hepatitis C and HIV^58^. Hence, targeting specific enhancers regulating *BACE1* and cell cycle genes could be a novel therapeutic avenue for AD, applicable to both early and late stage AD.

## Methods

No statistical methods were used to predetermine sample size.

### Study cohort

Prefrontal cortex tissue of our sample cohort was obtained from the MRC London Neurodegenerative Diseases Brain Bank with approval from the ethics committees of the Van Andel Research Institute and the Centre for Addiction and Mental Health (IRB #15025). For each individual, we had information on patient demographics (age, sex, and ethnicity), tissue quality (postmortem interval and brain pH), age of disease onset, and neurofibrillary tangle burden (Braak stage). Our study included 101 individuals (Female: 47; Male: 54), which were all of European ancestry. The mean age at death is 83.03 years; range was 54–105. These individuals displayed no/mild (Braak stage 1–2, *n* = 38), moderate (Braak stage 3–4, *n* = 32) and severe (Braak stage 5–6, *n* = 31) AD pathology (Supplementary Table 1). Braak stages of disease propagation have been described as the transentorhinal stages 1–2: unaffected/clinically silent, limbic stages 3–4: incipient AD, and neocortical stages 5–6: fully developed AD^3^. In our sample cohort, clinical diagnosis of AD correlated closely with neurofibrillary tangle burden (Pearson’s coefficient *R* = 0.78; *p* < 10^−15^). Neurons of the prefrontal cortex were selected for this study, as this region typically exhibits neuronal loss late in AD and is relevant to multiple common neuropathological phenotypes in the aging population^2^.

### DNA methylation fine-mapping with bisulfite padlock probes

DNA methylation was examined with single nucleotide resolution in human brain enhancers using the bisulfite padlock probe technique^59,60^ in prefrontal cortex neuronal nuclei (isolation described in Supplementary Methods). Enhancers were identified using EpiCompare tool^61^, which predicts tissue/cell-type-specific enhancers/promoters based on chromatin state data defined by ChromHMM tool from the RoadMap Epigenomics Project^13^. We used the 18-state ChromHMM model and included enhancers that are genic, active, weak, or poised/bivalent (7_EnhG1, 8_EnhG2, 9_EnhA1, 10_EnhA2, 11_EnhWk, 15_EnhBiv). We identified enhancers significantly enriched in the adult brain relative to all other body tissues, as determined by Fisher’s exact comparisons of 200 bp genome windows (ranked *p*-value corrected for multiple testing by FDR). They can be downloaded from the Tissue Specific Enhancers website (http://epigenome.wustl.edu/TSE/browse.php). In addition to including these brain-specific enhancers, we included all enhancers present in the adult prefrontal cortex (E073), inferior temporal lobe (E072), and substantia nigra (E074). Since promoters can at times act as enhancers^28^, we included brain promoters defined by the 18-state ChromHMM model to be active, near a TSS site, or poised/bivalent (1_TssA, 2_TssFlnk, 3_TssFlnkU, 4_TssFlnkD, 14_TssBiv; for E073, E072, and E074).

Padlock probes were generated to target the unique (non-repetitive) genome following bisulfite conversion using the ppDesigner software^59^. Probes (*n* = 59,009) were designed for both the forward and reverse DNA strands using the human GRCh37/hg19 genome. Probe sequences are described in Supplementary Data 5. Probes were synthesized using a programmable microfluidic microarray platform (CustomArray, Inc.) and were prepared for padlock investigations, as described^60^.

Fine-mapping of DNA methylation at enhancers using the bisulfite padlock probes approach was performed using the described protocol^60^. In brief, genomic DNA for each sample was bisulfite-converted and purified using the EZ DNA Methylation-Lightning Kit (Zymo Research). The bisulfite-converted DNA (140 ng) was hybridized to the padlock probes (1.5 ng). Targeted regions were extended using PfuTurbo C_x_ (Agilent Technologies) and circularization was completed using Ampligase (Epicentre). Non-circularized DNA was digested using an exonuclease cocktail and the remaining circularized DNA was amplified using a common linker sequence in the padlock probe. Libraries were PCR amplified, pooled in equimolar amounts, purified by QIAquick Gel Extraction kit (Qiagen) and quantified by qPCR (Kapa Biosystems) on a ViiA 7 Real-time PCR system (Applied Biosystems). Next-generation sequencing of the libraries was done across three flow cells (24 lanes) on an Illumina HiSeq 2500 machine in HiOutput mode at the Epigenetics Lab at the Centre for Addiction and Mental Health in Toronto, Canada, which yielded ~40 million reads/samples.

### Epigenomic data analysis

To analyze the bisulfite padlock probes data we used a custom pipeline based on the Bismark tool^62^. DNA methylation status was interrogated at every cytosine site (CpG and CpH) covered by padlock probes targeting 35,288 regulatory regions across the genome of 131 samples (*n* = 106 unique samples, two whole-genome amplified (WGA) control samples and 23 replicate samples). For each sample, adapter sequences were removed from the reads using Trimmomatic-0.32, and reads aligning to the phiX DNA spiked-in were removed. Reads were then aligned to the target reference genome (GRCh37/hg19). Methylation calls were determined as the percentage of fraction of spanning reads that retained the reference “C”, and were not converted to “T” from the bisulfite treatment. Methylation calls were only considered if 30 or more reads spanned the cytosine. Bisulfite conversion efficiency was on average 99.24 ± 0.007%. Technical and sequencing replicates confirmed a high reproducibility in sample-level methylation correlation analysis (average R for technical replicates: 0.976; average R for sequencing replicates: 0.998; Supplementary Fig. 2). There were seven samples (five unique samples and two replicates) that were excluded from further analyses due to poor inter-sample correlations (>10% difference). Replicate samples (*n* = 21) were then merged by taking the mean across replicates at each CpG/CpH site. All CpG/CpH sites with a methylation call in at least 70% of samples proceeded in the analyses. CpG/CpH sites that had a methylation call of 0 across all samples were excluded. We also removed cytosine sites overlapping common SNPs (minor allele frequency ≥ 0.05), as identified by the 1000 Genomes Project (phase 3 v5a 20130502 release for chr1~chr22, v1b 20130502 for chrX; all populations and European populations)^63^. At the end of these pre-processing steps, a subset of 101 samples with quality-controlled genome-wide methylation data at 1,207,507 CpGs/CpHs located across 29,132 regulatory regions were retained for downstream analysis.

Our analysis identified, in neurons, genomic regions with DNA methylation changes associated with AD pathology. We first transformed DNA methylation B-values to M-values using the lumi (v2.30.0) package, and then ran a multivariate robust linear regression model with empirical Bayes from limma (v3.30.13) statistical package^64^ using Braak stage as the independent variable and each CpG/CpH methylation as an dependent variable, adjusting for age, sex, postmortem interval, and neuron subtype proportion (see neuronal subtype deconvolution methods below). We then identified differentially methylated regions in AD neurons, by determining the regions enriched with CpG/CpH methylation differences in AD. First, the top 10% significant CpGs/CpHs were selected based on the linear regression modeling results as the differentially methylated Cs (DMCs). Regions for DMCs were generated by grouping all cytosines within ±1000 bp. A hypergeometric test was carried out to assess the enrichment of DMCs in each region relative to all cytosines profiled in each of the same regions (DMCs and non-DMCs), with the background as top 10% of cytosines relative to all profiled cytosines. Benjamini-Hochberg adjustment for multiple testing was then applied. Differentially methylated regions (regions enriched with top 10% DMCs) met the criteria of *q* < 0.05 and ≥1% cytosine methylation change between Braak stage groups. The same approach was used in the analysis identifying differentially methylated regions in early AD pathology (Braak stage 1–4). We also confirmed that there was no inflation (genomic inflation factor = 0.90) or over-detection bias (test statistic bias = −0.10) in our data using BACON^65^ (Bayesian correction method), and that inflation/bias adjustment yielded analogous sites (Pearson’s correlation *R* = 0.95, *p* < 10^−15^).

The major neuronal subtypes in the prefrontal cortex consist of 70–85% excitatory glutamatergic neurons, while the remaining 15–30% are inhibitory GABAergic neurons^66^. In our DNA methylation analysis in prefrontal cortex neurons, we controlled for variation in neuronal subtype (glutamate and GABA) between samples. Neuronal subtype proportion for each sample was quantified using the CETS tool^67^ and markers obtained from DNA methylation reference maps generated for glutamate and GABA neurons by PsychENCODE. First we selected 1144 reference cytosine sites that were both present in our epigenomics dataset and showed significant differences in DNA methylation between isolated glutamate and GABA neurons in the EpiGABA methylation dataset from PsychENCODE Knowledge Portal (Synapse ID: syn4874178 [https://www.synapse.org/#!Synapse:syn4874178]). Reference cytosine sites met the following criteria: paired *t*-test *p* ≤ 0.05 between neuronal subtypes; DNA methylation change ≥5%, and not significantly different in AD. We then used CETS^67^ and the reference cytosine sites to estimate neuronal subtype proportion in each sample of our epigenomics dataset (Supplementary Fig. 3a).

In addition, we verified that there was not a selective loss of any of the 21 specific types of glutamatergic or GABAergic neurons^29^. For this, we used the gene body CpH methylation markers provided in a recent single cell DNA methylome analysis in the human frontal cortex^29^. In our dataset, we averaged CpH methylation within gene bodies (±100 kb), and found 582 neuronal gene signatures (out of 1012) reported in ref. ^29^ Cell-type deconvolution was performed using CIBERSORT^68^ (http://cibersort.stanford.edu), which has previously been applied to DNA methylation data for cell-type deconvolution^69^. Using the neuronal signature matrix (582 gene CpH markers), CIBERSORT^68^ was run (100 permutations). We observed that the proportion of the specific neuronal subtypes in our NeuN+samples was analogous to those previously reported^29^, and there was no significant differences in any type of neuron between the Braak stage groups (Supplementary Fig. 3b).

TF analysis was performed using oPOSSUM (http://opossum.cisreg.ca/oPOSSUM3/). Sequences of the 1224 enhancer regions significantly associated with AD pathology was the input and the sequences of all regions assessed in the bisulfite padlock probe assay was the background. To identify a significantly over-represented TF families at AD enhancer regions, we performed the TF Binding Site (TFBS) Cluster Analysis in oPOSSUM.

### Gene annotation and enrichment analysis

Since enhancers dynamically regulate gene expression through three-dimensional physical interactions, we analyzed Hi-C data generated in from the human prefrontal cortex^33^ (Illumina HiSeq 2000 paired-end raw sequence reads; *n* = 1 sample; 746 Million reads; accession: GSM2322542 [https://www.ncbi.nlm.nih.gov/geo/query/acc.cgi?acc=GSM2322542]). Our Hi-C analysis pipeline involved Trim Galore (v0.4.3) for adapter trimming, HiCUP (v0.5.9; https://www.bioinformatics.babraham.ac.uk/projects/hicup/) for mapping and performing quality control, and HOMER^70^ for identifying significant interactions (default: *p* < 0.001 and *z* score > 1.0), with a 40 kb resolution. Our analysis identified 58,758 interactions in total; 951 interactions involving regions targeted in our bisulfite padlock probe assay. Hi-C gene annotation involved identifying interactions with gene promoters, defined as ±2 kb of a gene TSS. We then generated a list of genes affected by the differentially methylated enhancers in AD (list of distinct genes, where each gene is counted only once). There were 1207 genes annotated for differentially methylated regions in AD, while 5893 genes were determined for the background (total possible number of genes based on all regions captured in our padlock data). To account for proximal interactions, we use the GREAT software (http://great.stanford.edu/public/html/) to further identify nearby genes, which added 1224 genes proximal to the differentially methylated enhancers and 4206 to the background. In total, we identified 2431 genes associated with the differentially methylated regulatory regions in AD (10,099 genes determined as the background).

Pathway enrichment analysis for the genes affected by the AD enhancers was done using MetaCore (https://clarivate.com/products/metacore/) and was relative to our background genes. Proteomics data were obtained from a published proteomic analysis of neurons microdissected from Alzheimer’s disease cortex tissue^36^.

We examined whether the enhancer regions associated with AD were overlapping or near genetic polymorphisms (SNPs) identified in a large GWAS meta-analysis of AD (International Genomics of Alzheimer’s Project, IGAP^37^). First, we extracted all 1457 SNPs (GWAS *p* < 10^−8^) from IGAP (these 1457 SNPs are in linkage disequilibrium to the 21 tagSNPs significantly associated with AD in the IGAP study). Then using FunciSNP^71^, we found all other additional SNPs in linkage disequilibrium with the 1457 SNPs with *R*^2^ ≥ 0.8, using 1000 Genomes phase 3 (http://www.internationalgenome.org/) as reference. Enhancer overlap/proximity to a total of 1631 SNPs was examined. We also assessed whether the genes targeted by the AD enhancers (as determined in Hi-C analysis) were among the identified genes in the GWAS meta-analysis of AD^37^.

We asked whether there was a relationship between the entire AD GWAS and our epigenomic study of enhancers in AD neurons by pairing cytosines to the nearest SNPs located in the same LD block. To do this, we calculated LD blocks (1000 Genomes EUR population) using plink (http://zzz.bwh.harvard.edu/plink/), and then assigned each HapMap3 SNP the *p* value and *b* value of the closest CpG or CpH, with a requirement that the cytosine and SNP shared the same LD block. We also obtained the computed LD Scores (determined by LDSC^72^, calculated based on 1000 Genomes EUR population). We then performed a simple linear regression correlating the AD GWAS (IGAP)^37^ and our assigned SNP values from the epigenomics study, adjusted for LD score.

### RNA-sequencing

We used RNA-seq to profile the mRNA transcriptome in the prefrontal cortex of individuals with no/mild (Braak stage 1–2, *n* = 10 individuals), moderate (Braak stage 3–4, *n* = 9), and severe (Braak stage 5–6, *n* = 6) AD pathology (samples also in DNA methylation study above). Frozen brain tissue was pulverized by CryoPREP (Covaris Inc.), and 20–30 mg of pulverized frozen tissue was lysed and homogenized in QIAzol (Qiagen) using the TissueLyser II (Qiagen). Total RNA were immediately extracted using the RNeasy Universal Mini Kit (Qiagen) including DNase digestion. Resulting RNA quantity was assessed by Nanodrop 8000 (Thermo Scientific) and quality was assessed with an Agilent RNA 6000 Nano Kit on a 2100 Bioanalyzer (Agilent Technologies, Inc.). Libraries were prepared by the Van Andel Genomics Core from 500 ng of total RNA using the KAPA RNA HyperPrep Kit with RiboseErase (v1.16) (Kapa Biosystems). RNA was sheared to 300–400 bp. Prior to PCR amplification, cDNA fragments were ligated to Bioo Scientific NEXTflex Adapters (Bioo Scientific). Quality and quantity of the finished libraries were assessed using a combination of Agilent DNA High Sensitivity chip (Agilent Technologies, Inc.), QuantiFluor dsDNA System (Promega Corp.), and Kapa Illumina Library Quantification qPCR assays (Kapa Biosystems). Individually indexed libraries were pooled and 75 bp, single end sequencing was performed on an Illumina NextSeq 500 sequencer using a 75 bp HO sequencing kit (v2) (Illumina Inc.), with all libraries run across 2 flow cells. Base calling was done by Illumina NextSeq Control Software v2.0 and output was demultiplexed and converted to FastQ format with Illumina Bcl2fastq v1.9.0.

Trim Galore (v0.11.5) was used to trim the 75 bp single ends reads prior to genome alignment. STAR (v2.3.5a) index was generated using Ensemble GRCh37 p13 primary assembly genome and the Gencode v19 primary assembly annotation. Read alignment was performed using a STAR and counted using RSEM. Gene counts matrix was imported into R (v3.4.1) and low expressed genes (counts per million <1 in more than 30% of samples) were removed prior to trimmed mean of M-values normalization in EdgeR (v3.16.5). The limma (v3.30.13) statistical package^64^ was used to transform the count matrix to log2-counts per million and fit a robust linear model examining AD Braak stage and adjusting for age, sex, postmortem interval, neuronal cell composition, and RNA Integrity Number (RIN) with empirical Bayes. The variancePartition package^73^ was used to quantify variation of each factor in RNA-Seq data (Supplementary Fig. 7). Differential expression *p*-values were adjusted for multiple testing correction using the Benjamini-Hochberg method in the stats package (v3.3.3). Statistical significance for differentially expressed genes was *q* < 0.05.

Our RNA-seq analysis is corrected for the proportion of neuronal cells in each sample. Cell-type deconvolution was performed using CIBERSORT^68^ (http://cibersort.stanford.edu), which performs a linear support vector machine learning algorithm on normalized cell-type-specific count data. In this approach, we used a gene signature matrix (involving 903 cell-specific marker genes) derived from single cell RNA-seq measures in adult human brain cells (signature matrix;^74^ source^75^). CIBERSORT^68^ was run (100 permutations), and values were used for the neuronal cell composition adjustment in the robust linear model for differential expression analysis.

In addition, RIN was measured and verified by transcript integrity number TIN^76^ using human representative RefSeq transcripts to determine the 5′ to 3′ integrity of mRNA within each sample. This approach determines both RNA quality based on the integrity of mRNA and accounts for transcript specific decay (rather than an indirect measure of total/ribosomal RNA)^76^.

### Network analysis

Using the OmicsIntegrator^77^, we merged epigenetic and transcriptomic sequencing data to identify underlying molecular pathways in AD. The OmicsIntegrator^77^ package consists of two software tools: Garnet and Forest^77^. The Garnet algorithm identified TFs associated with mRNA expression changes by incorporating epigenetic data (epigenetically altered sequences scanned for TF binding sites), and then TF affinity scores were regressed against gene expression changes^77^. We used the 1224 significantly epigenetically altered enhancer regions in AD and their related sequences, as well as the significantly differentially expressed genes in AD in our RNA-seq experiment and log fold change, as input for Garnet. Garnet confirmed that a top differentially expressed motif was the ETS family (*p* < 0.05), as described earlier. Next, the Forest tool was used to find functional subnetworks connecting omic hits. Here, a confidence-weighted interactome (iRefIndex protein-protein interaction network, version 13.0) is integrated with our data using the Steiner forest algorithm^77^. To identify AD subnetworks (Fig. 3), we used our Garnet output along with differentially expressed genes in AD with the prize score (as calculated as −log2(*q* value)), as input for Forest. We repeated this step adding the independently generated AD proteomics data^36^ (Supplementary Fig. 8). Forest output was visualized with Cytoscape (v3.6.0).

### Aging analysis

Our analyses of DNA methylation changes with age were performed on CpG (*n* = 531) and CpH (*n* = 3,661) sites located in top differentially methylated enhancers in AD (*n* = 848 enhancers with both epigenetic and associated gene transcript differences in AD, as identified in omics integration analysis). The % methylation was calculated from number of methylated sites/total number of sites as described previously^27^. A linear regression (limma) was performed to determine the % CpH methylation change occurring with age, after controlling for sex, postmortem interval, Braak stage, and neuron subtype proportion. Within each Braak stage group the % methylation change with age was determined by linear regression (limma), adjusted for sex, postmortem interval, and neuronal proportion. We also examined aging changes in infancy and early adulthood in all differentially methylated enhancers in AD using a whole-genome bisulfite sequencing dataset of isolated neurons^27^.

The DNA methylation age calculator^17^ was used to determine whether the epigenetic age of AD neurons exceeded their chronological age. CpH methylation in enhancers for Braak stage 1–2 (*n* = 38 individuals) was used as the training dataset to estimate the DNA methylation age in the test datasets: Braak stage 3–4 (*n* = 32) and Braak stage 5–6 (*n* = 31). Using the training dataset, we regressed a calibrated version of chronological age on 3661 CpH methylation using an elastic net regression model^17^. As done previously^17,78,79^, the elastic net mixing parameter, alpha, was set to 0.5, and the lambda parameter was determined through a tenfold cross validation of the training data. Next, using the resulting model and 3661 CpH methylation sites, the DNA methylation age was predicted for each test subject. A paired *t*-test was performed to determine if there was a significant change between the chronological age and DNA methylation age in the test datasets.

### URLs

NIH Epigenomics Roadmap, http://www.roadmapepigenomics.org/data/; Tissue Specific Enhancers; 1000 Genomes Project, http://www.internationalgenome.org/; European Nucleotide Archive; PsychENCODE Knowledge Portal: https://www.synapse.org/#!Synapse:syn4921369; MetaCore; CIBERSORT: http://cibersort.stanford.edu; plink; GREAT: http://great.stanford.edu/public/html/; oPOSSUM; HiCUP.

### Reporting summary

Further information on research design is available in the Nature Research Reporting Summary linked to this article.

## Supplementary information

Supplementary Information

Description of Additional Supplementary Files

Supplementary Data 1

Supplementary Data 2

Supplementary Data 3

Supplementary Data 4

Supplementary Data 5

Reporting Summary

## Data Availability

All sequencing data generated in this study are available from the NCBI Gene Expression Omnibus (GEO) database under the accession number GSE110732. Custom code for DNA methylation and RNA-seq analysis is available at https://github.com/lipeipei0611/AD_Enh.
