# Supplementary material for: Epigenetic dysregulation of enhancers in neurons is associated with Alzheimer’s disease pathology and cognitive symptoms
Source: Nat Commun. 2019 May 21;10:2246. doi: 10.1038/s41467-019-10101-7 (PMC6529540; doi:10.1038/s41467-019-10101-7)
Supplement: Supplementary file 1 — Supplementary Information [file 41467_2019_10101_MOESM1_ESM.pdf]

# Epigenetic dysregulation of enhancers in neurons is associated with Alzheimer's disease pathology and cognitive symptoms

Li P., et al.

**This PDF file includes:**

Supplementary Figure 1-12

Supplementary Tables 1-8

Supplementary Methods

Supplementary References

**Other Supplementary Material for this manuscript includes the following:**

Supplementary Data 1-5

## Supplementary Figures and Figure Legends

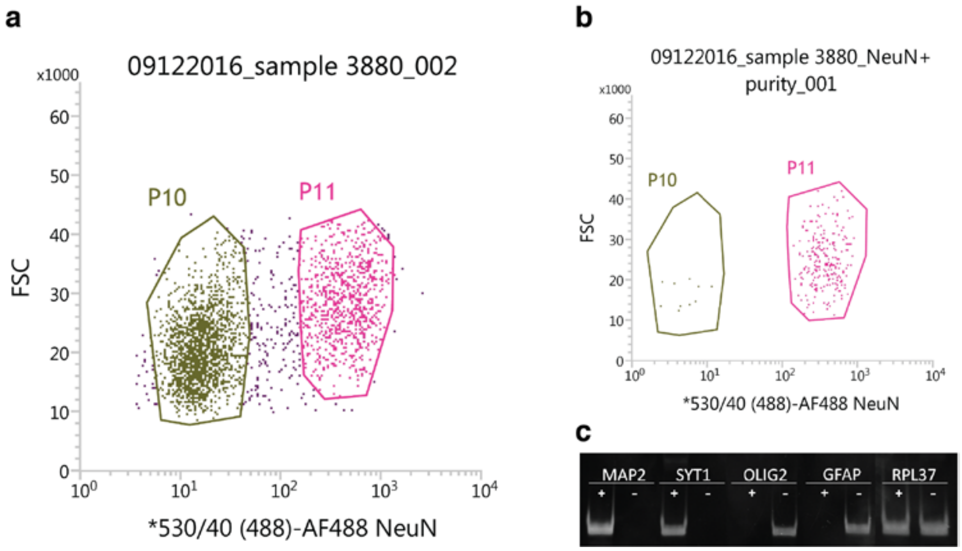


**Supplementary Figure 1.** Isolation of human prefrontal cortex neurons by flow cytometry. (**a**) Representative gating image for flow cytometry sorting of neuronal (NeuN+) and glial/non-neuronal (NeuN–) nuclei, after gating for 7-AAD positive nuclei. (**b**) Reanalysis of NeuN+ aliquots after sorting show a high purity for NeuN+ stained nuclei (on average 96%). (**c**) mRNA-qPCR analysis confirming the purity of our neuronal and glial isolations. MAP2 and SYT1 are neuronal markers, whereas OLIG2 and GFAP are glial markers (+ and – refers to NeuN+ and NeuN–, respectively). The control RPL37 is located in both neurons and glia. Analysis of an aliquot of 1,000 NeuN+ and NeuN– nuclei prepared in RNase inhibitor (Superase, Thermo Fisher Scientific), and cDNA conversion and qPCR amplification done with the Power SYBR Cells-to-CT kit (Thermo Fisher Scientific).

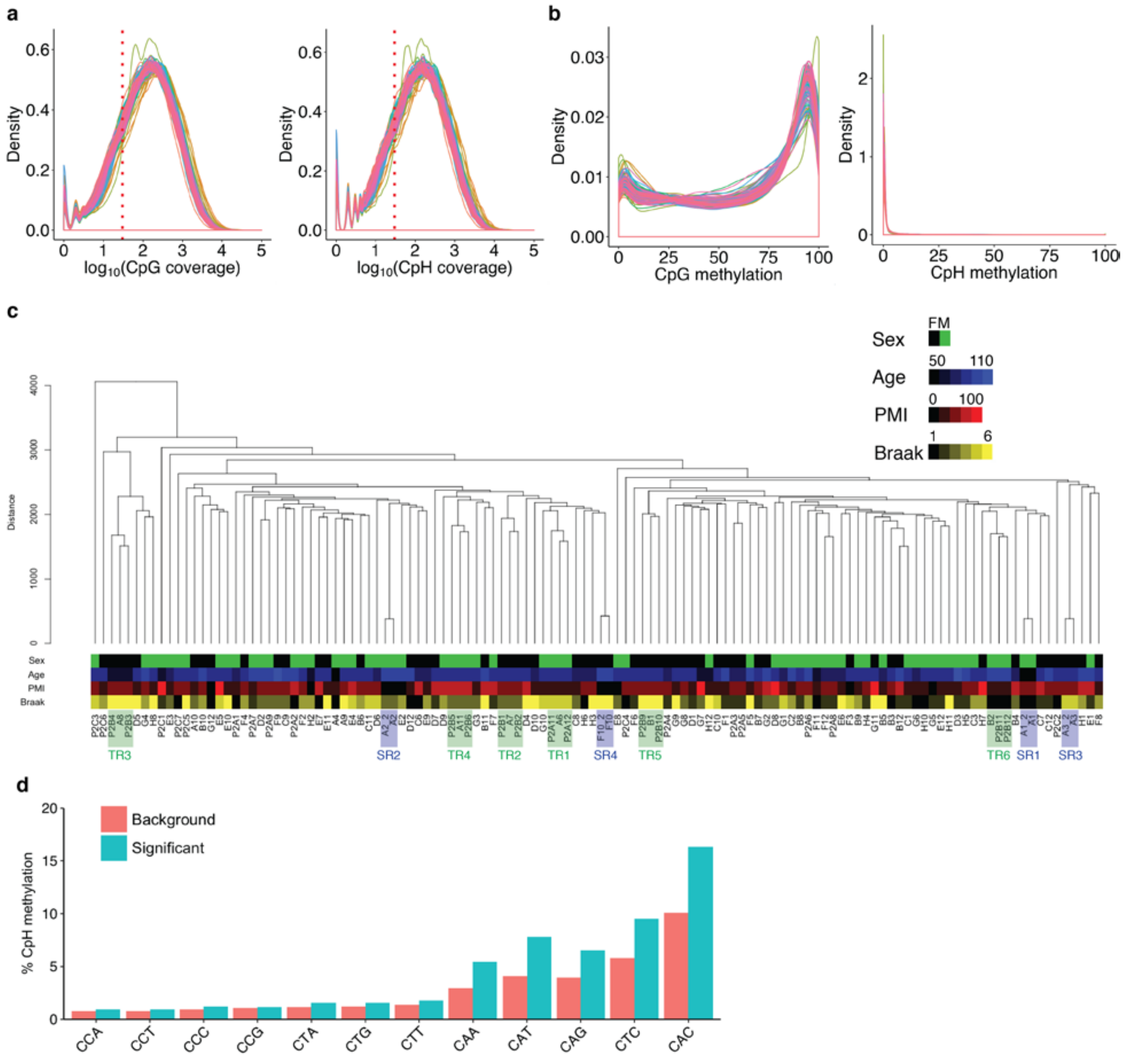


**Supplementary Figure 2.** Analysis of cytosine site coverage and sample distributions showing high technical reproducibility. (**a**) Density plots showing read coverage at CpG (left) and CpH (right) for each individual sample. Red dotted line shows 30× coverage. (**b**) Density plots showing methylation distribution at CpG (left) and CpH (right) with ≥30× coverage for each sample separately. (**c**) Dendrogram showing clustering of samples based on correlation of DNA

methylation. Pearson's correlation of methylation for the 10,000 most variable CpG and CpH sites. Technical replicates (TR, library preparation replicated for select DNA samples) and sequencing replicates (SR, sequencing replicated across lanes/flow cells for select libraries) are shown in green and blue colors, respectively. The average correlation scores for SR is 0.998; for TR is 0.976; for all the other unique biological samples is 0.967, as determined by Pearson's correlation of all CpG and CpH methylation sites. (**d**) Averaged % methylation for each type of CpH site across all samples. Both significant differentially methylated Cs in AD and 1.2M background Cs are shown. Enhancer regions in neurons in this study had a similar distribution of CpH methylation to previous whole genome studies in neurons[1].

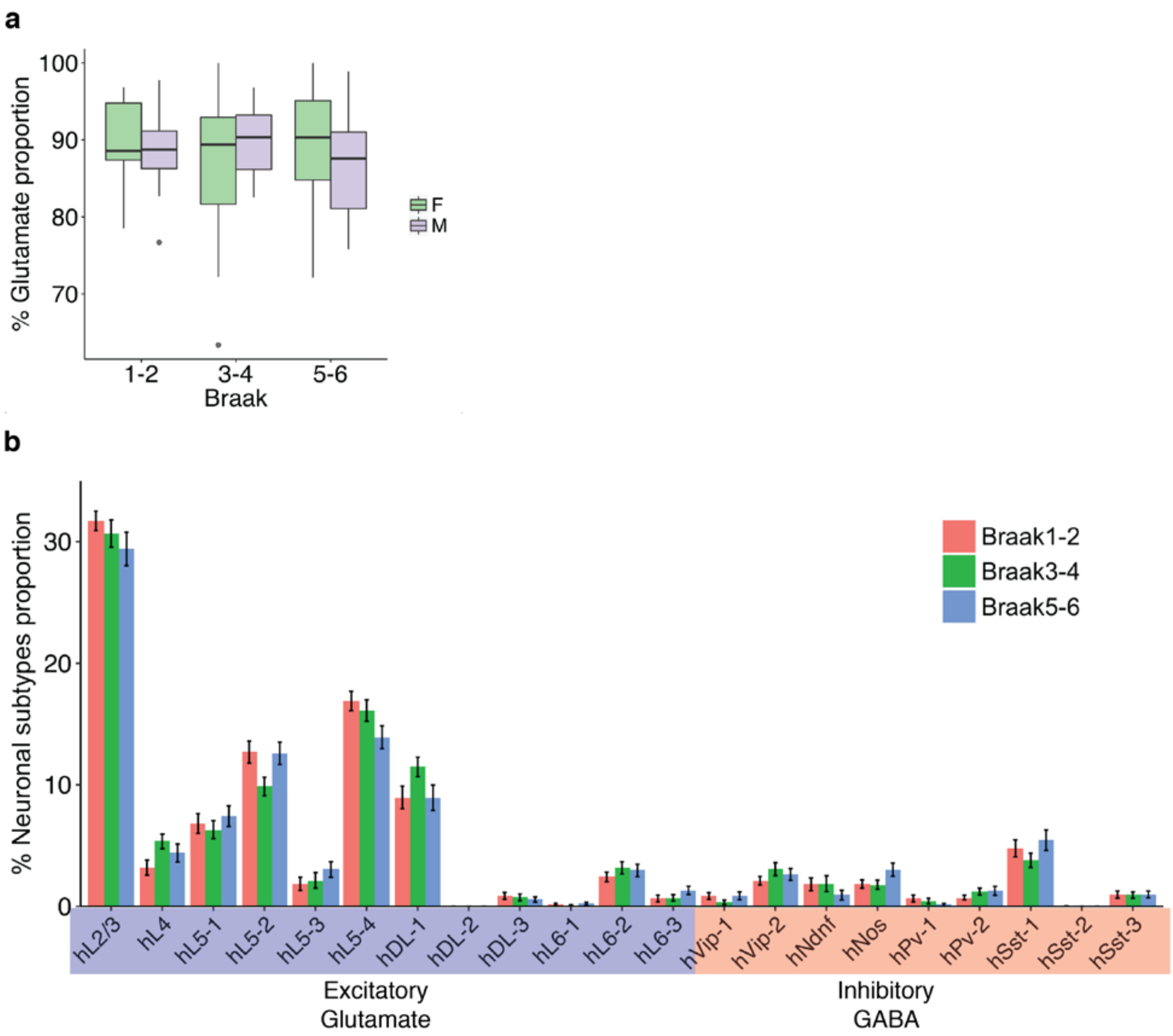


**Supplementary Figure 3.** Neuronal subtypes in the neuronal nuclei fraction (NeuN+) isolated from the prefrontal cortex of individuals with no/mild, moderate, and severe AD pathology (Braak stage 1-2, 3-4, and 5-6, respectively). (**a**) Proportion of glutamate neurons in each of the Braak stage groups. The relative abundance of glutamate vs GABA neurons in the NeuN+ fraction was calculated by neuronal subtype deconvolution, using neuron subtype specific markers (n=1,144 cytosine sites). This analysis indicated that the majority of neuronal nuclei isolated from the prefrontal cortex were glutamatergic (88.56%). There was no difference in glutamate proportion between the different Braak stage groups or between sexes, as determined

by 3-way ANOVA. The average percentage of glutamate proportion for Braak 1-2 is 0.892; Braak 3-4 is 0.885; Braak 5-6 is 0.878. The boxplot center line is the median, the lower and upper limits are the first and third quartiles ($25^{th}$ and $75^{th}$ percentiles), and the whiskers are 1.5× the interquartile range. (**b**) Proportion of 21 specific types of neurons in NeuN+ fractions of Braak stage groups. Each specific neuronal type is averaged for each Braak stage group. Glutamatergic neuronal types highlighted in blue and GABAergic neuronal types highlighted in pink. Averaged % neuronal subtype proportion ± s.e.m. Neuronal proportions were similar to as previously described[1], and there was no significant differences between Braak stage groups for any of the specific neuronal subtypes.

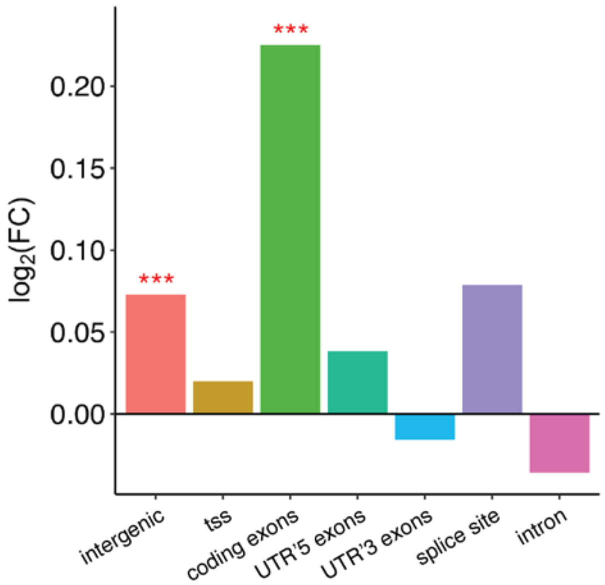


**Supplementary Figure 4.** Genomic locations of neuronal enhancers and promoters significantly associated with AD pathology. $Log_2$(FC) refers to log fold change in enrichment of genomic location with differentially methylated enhancer/promoter cytosines. In AD, differentially methylated cytosines in enhancers/promoters were enriched in intergenic and coding exons. ***$p \leq 10^{-5}$, as determined by hypergeometric tests.

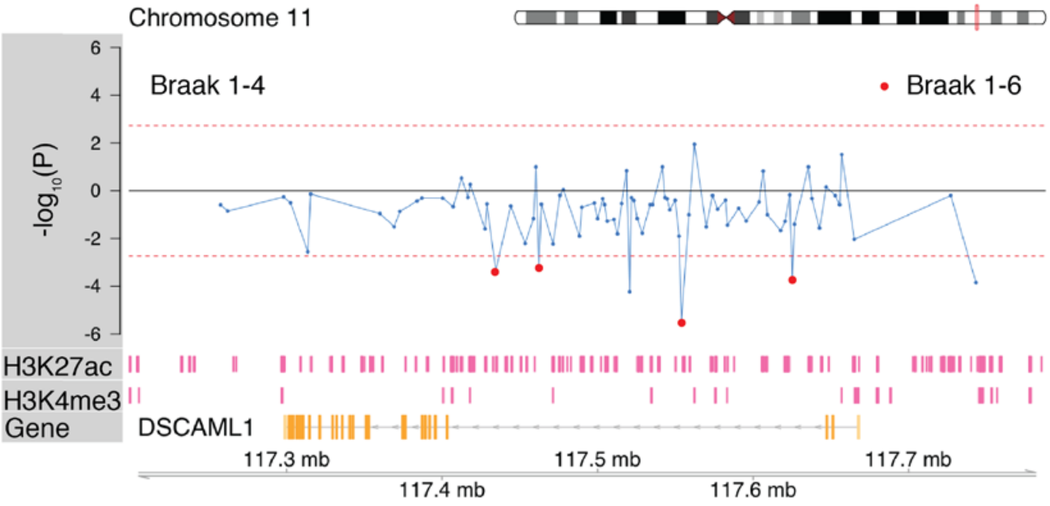


**Supplementary Figure 5.** *DSCAML1* enhancers exhibit DNA methylation changes that occur early in AD. In prefrontal cortex neurons, DNA methylation differences at enhancers and promoters were analyzed in individuals that did not yet have neurofibrillary tangle pathology in the prefrontal cortex (Braak stage 1-4). In *DSCAML1* intron 3 there were 5 sites with significant DNA methylation changes occurring prior to the arrival of tangle pathology. $-\log_{10}(P)$ refers to the significance of differentially methylated cytosine enrichment at enhancers, with the sign corresponding to the direction of methylation change (hyper or hypomethylated). Differentially methylated enhancers in advanced AD (Braak stage 1-6) are also noted by red dots. Tracks for neuronal (NeuN+) H3K27ac and H3K4me3, from PsychENCODE (pink, n=9 individuals) are shown. Threshold for genome-wide significance (dashed line) is $q<0.05$.

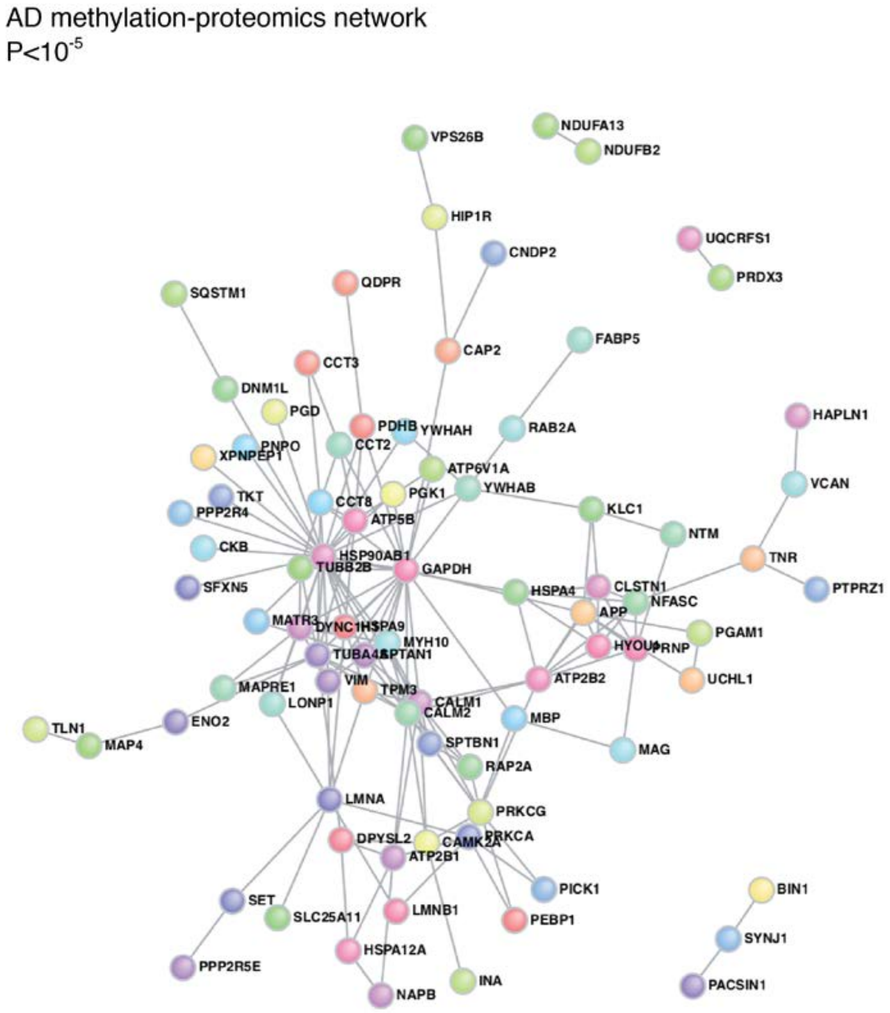


**Supplementary Figure 6.** Interaction network of proteins with differential regulation in AD neurons. Two independent datasets were overlapped: 1) gene targets of enhancers with DNA methylation abnormalities in AD cortical neurons, and 2) proteomic changes in laser captured cortical neurons in AD[2]. 109 genes had both a differentially methylated enhancer and proteomic abnormality in AD neurons (genes identified in the two datasets significantly overlapped, $p<10^{-5}$, hypergeometric test). Image showing protein-protein interaction network, as determined by DAPPLE, for proteins affected in AD neurons in both datasets (proteomic and epigenetic enhancer disruption). Network nodes represent proteins involved in AD, and edges represent

protein-protein associations. Protein-protein interactions are highly enriched ($p$<0.001, 1000 permutation test).

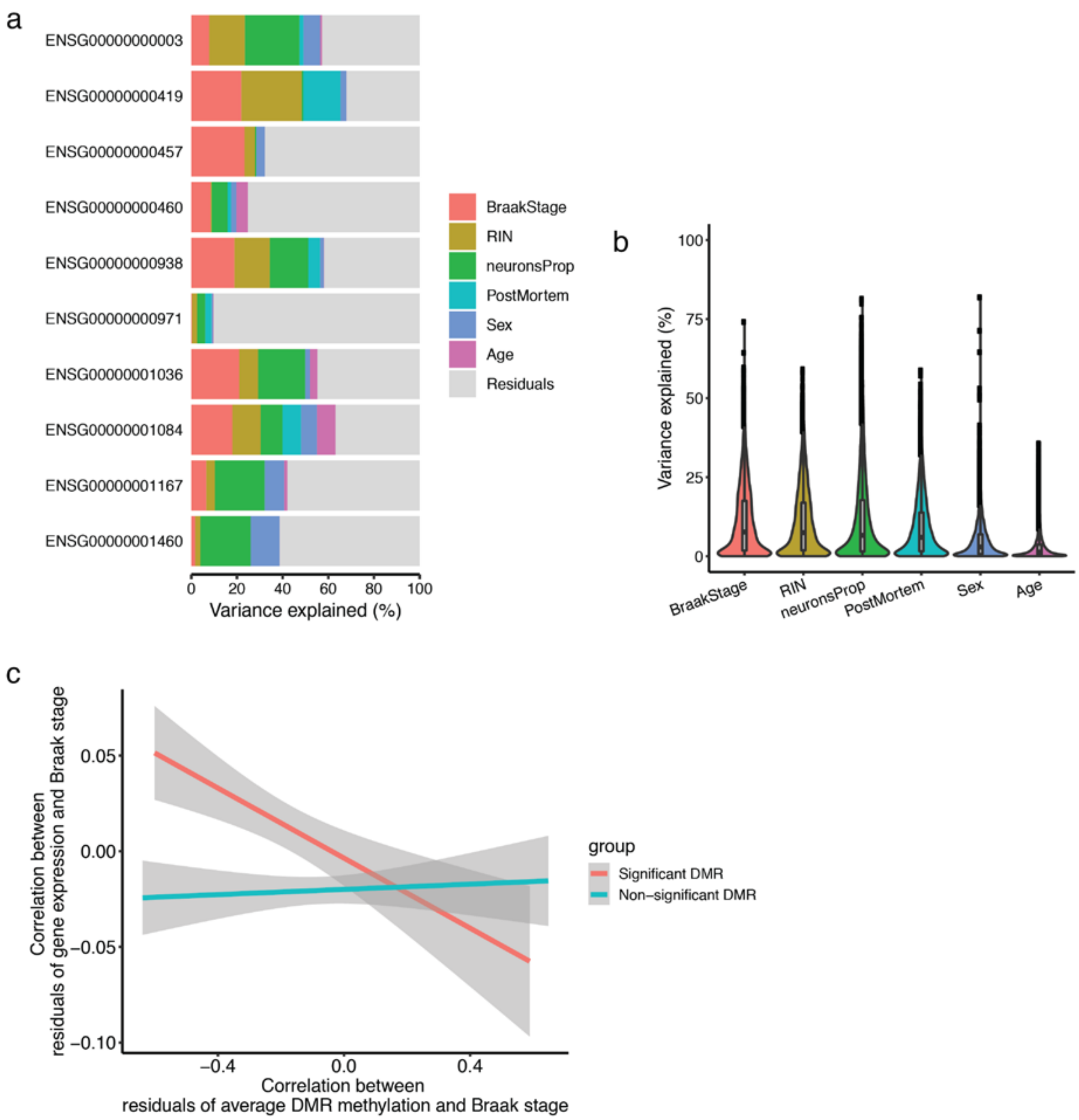


**Supplementary Figure 7.** The contribution of sources of variation in the RNA-Seq data. (**a**) Bar plot showing variance fractions for 10 example genes. (**b**) Violin plot depicting the contribution of each factor to total variance. On average 10.8% ± 0.078%, 10.6% ± 0.076%, 11.6% ± 0.097%, 8.9% ± 0.067%, 4.7% ± 0.039%, 2.8% ± 0.030% of total RNAseq variance was explained by Braak stage, RIN, neuronal proportion, postmortem interval, sex, and age, respectively (n=18,221 genes). The boxplot center line is the median, the lower and upper limits are the first

and third quartiles (25th and 75th percentiles), and the whiskers are 1.5× the interquartile range. (**c**) The relationship between enhancer DNA methylation changes and target gene expression changes with AD pathology. Our DNA methylation and corresponding RNA-seq data (n=25 individuals) was used to examine enhancers with DNA methylation status significantly (significant DMR) or non-significantly (non-significant DMR) associated with AD pathology. AD-associated enhancers exhibited DNA methylation changes with AD Braak stage that were inversely correlated to target gene expression changes with AD Braak stage ($p<0.001$, Pearson's correlation). AD-associated enhancers showed a greater relationship between DNA methylation and gene expression changes in AD, than enhancers not associated with AD ($p<0.001$, Fisher r-to-z transformation). Correlation line with 95% confidence interval shown of data for n=1,794 genes with significant enhancer DMRs and n=5,165 genes with non-significant enhancer DMRs.

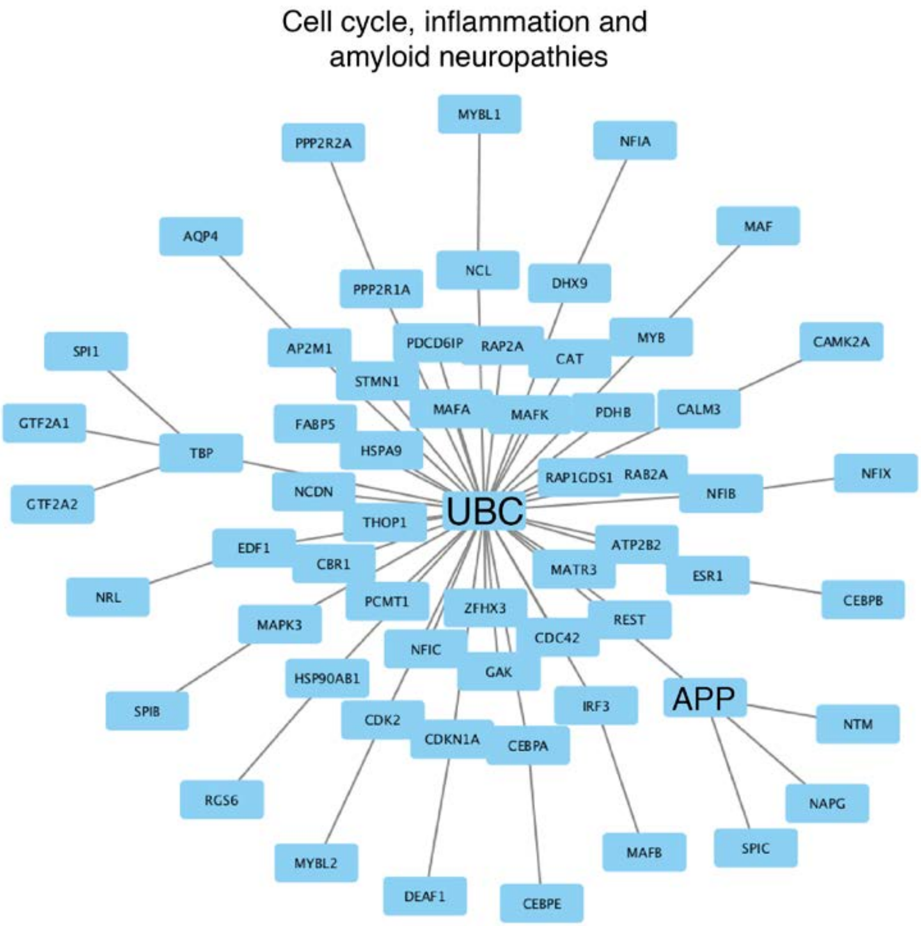


**Supplementary Figure 8.** Network analysis integrating epigenetic, transcriptomic, and proteomics data identifies pathways involved in AD. The OmicsIntegrator[3] was used to combine three omics datasets identifying changes in AD cortical neurons: differentially methylated enhancer data, differential gene expression data, and proteomics data. A high-confidence network relevant to AD was identified with *UBC* as the focal gene hub. Pathway analysis by MetaCore revealed a significant enrichment in cell cycle, inflammation, and amyloid neuropathies ($q<0.01$, hypergeometric distribution).

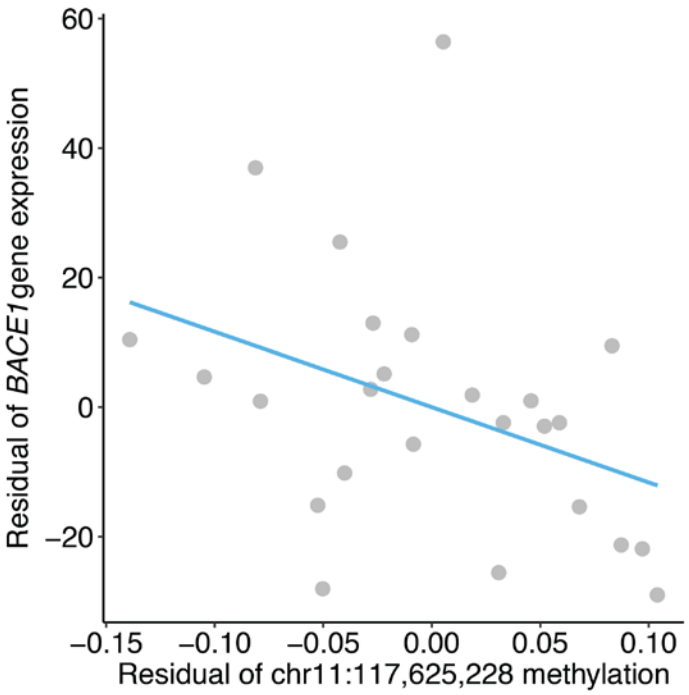


**Supplementary Figure 9.** Correlation between DNA methylation at cytosine site within enhancer targeting *BACE1* (chr11: 117,625,228) and *BACE1* mRNA levels. Analysis comparing DNA methylation status to *BACE1* mRNA expression was adjusted for sex, age, postmortem interval, neuronal proportion, and RIN. Enhancers that target *BACE1* in our Hi-C analysis were examined. The same enhancer with the cytosine (cg07533617; Fig. 4) from the ROSMAP analysis contained a site (chr11:117,625,228; 5.51% hypomethylation in AD) at which loss of DNA methylation was significantly associated with increased *BACE1* mRNA expression ($q$<0.05, robust linear regression). Correlation between residuals of DNA methylation (beta values at chr11:117,625,228, x-axis) and *BACE1* mRNA levels (CPM, y-axis) shown.

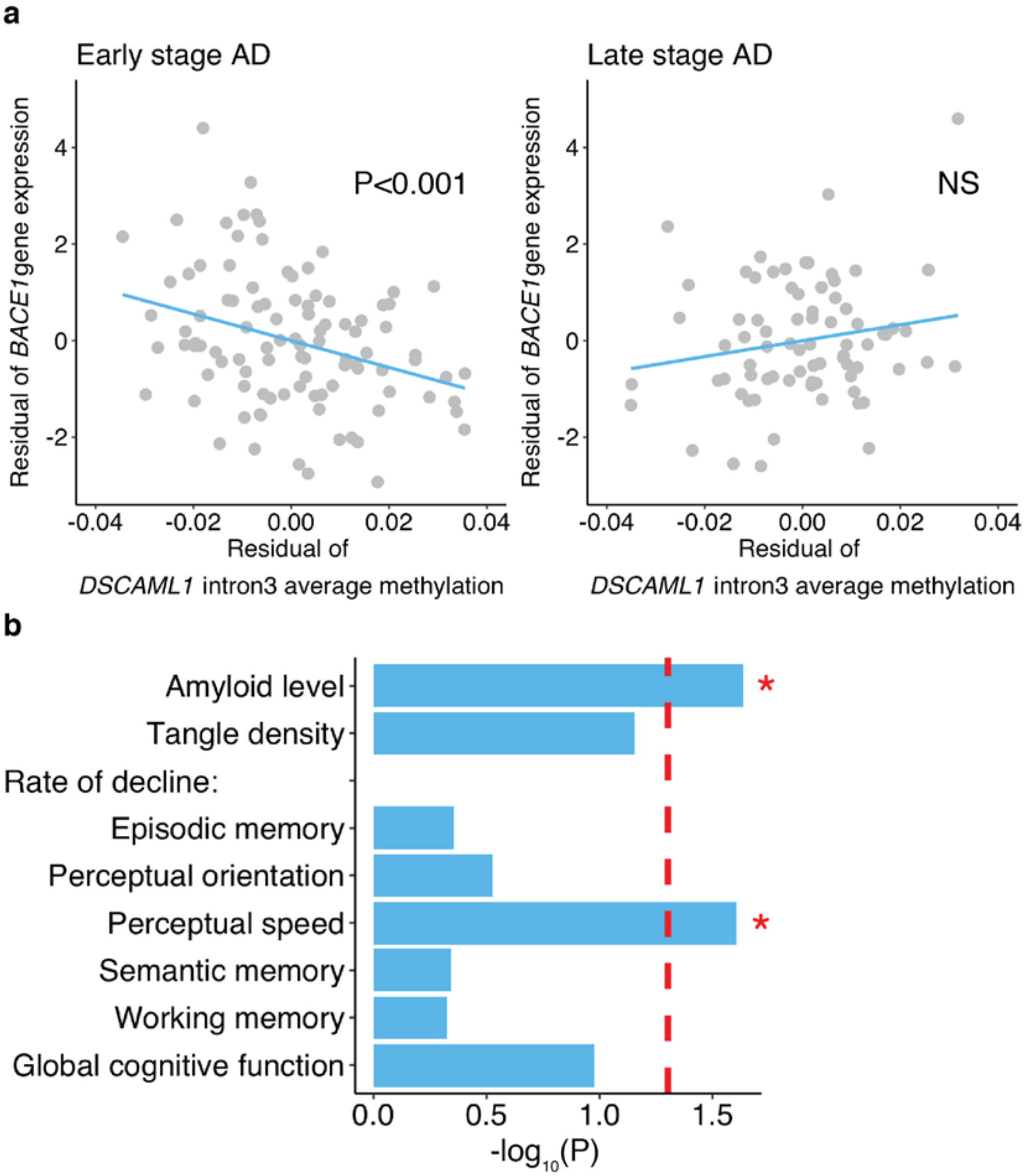


**Supplementary Figure 10.** DNA methylation at *DSCAML1* intron 3 is associated with *BACE1* gene expression in early disease, and to the pathology and cognitive symptoms of AD. (**a**) Correlation between DNA methylation at *DSCAML1* intron 3 (average of 22 CpG sites) and *BACE1* mRNA expression in the AD prefrontal cortex. Averaged *DSCAML1* intron 3

methylation was correlated with *BACE1* expression in the prefrontal cortex in early disease stages (Braak ≤4, $p<0.001$, robust linear regression model), but no longer in late stage AD (n=101 and 76 AD patients, respectively). Analysis performed using the ROSMAP dataset, which contained AD patients with both transcriptomic data generated by RNA-seq and genome-wide DNA methylation data generated by 450K Illumina DNA methylation arrays. (**b**) Significance of correlation between averaged DNA methylation at *DSCAML1* intron 3 and AD pathology/cognitive symptoms. Analysis used the ROSMAP DNA methylation and pathological/clinical data (n=465 AD and controls). DNA hypomethylation at *DSCAML1* intron 3 was significantly correlated with increased amyloid pathology and the decline in perceptual speed (*$p<0.05$, linear mixed model with annual cognitive measures as the longitudinal outcomes and DNA methylation as the predictor). (**a, b**) All analyses of ROSMAP data are adjusted for sex, age, postmortem interval, years of education, neuronal cell proportion, plus in (**a**) RIN.

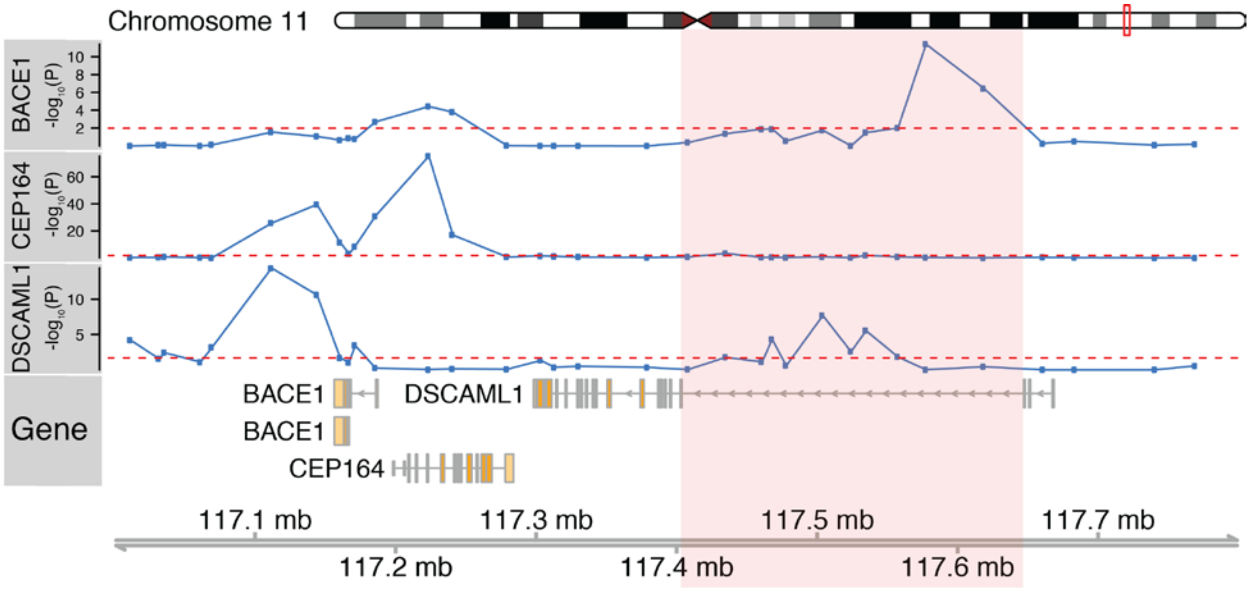


**Supplementary Figure 11.** The effect of genetic variation within *DSCAML1* intron 3 enhancers on transcript levels of nearby genes (*BACE1*, *CEP164*, and *DSCAML1*) in the prefrontal cortex (n=278 AD and control individuals in ROSMAP dataset). Analysis of haplotype association with gene expression that controlled for age, sex, postmortem interval, years of education, Braak stage, RIN, and neuronal proportion, was performed on the genomic area surrounding *DSCAML1* intron 3 (chr11:117,000,000-117,800,000, genomic area centered on *DSCAML1* intron 3; n=34 haplotypes identified using 4,305 SNPs). Each blue track shows the significance of the haplotype association with gene expression (dashed red line is $q<0.05$).

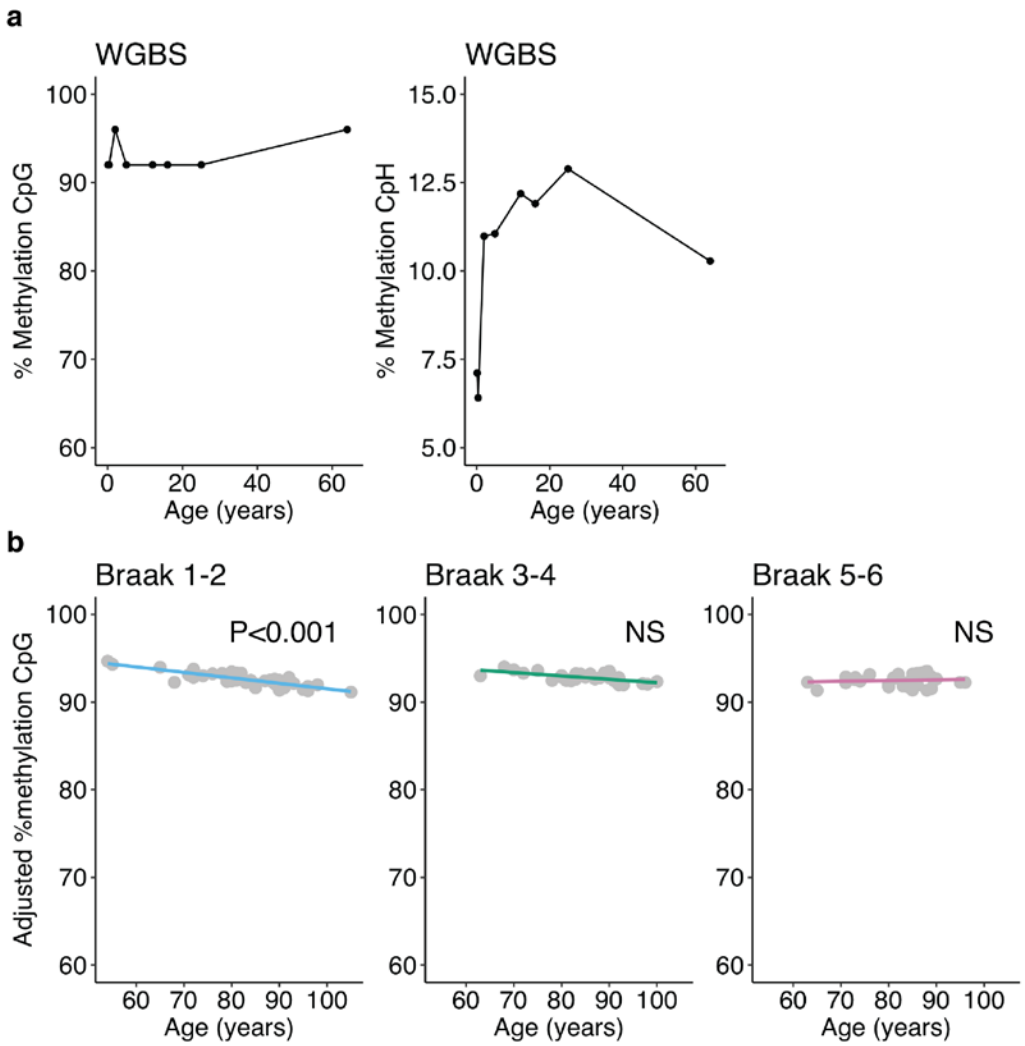


**Supplementary Figure 12.** CpG and CpH methylation profiles at enhancers in aging neurons. (**a**) % CpG (left) and CpH (right) methylation status from infancy to adulthood in healthy neurons. Methylation status at CpG and CpH sites in enhancers relevant to AD were examined in whole-genome bisulfite sequencing data from ref[4], with a 1× methylated read threshold (n=1 individual per age point). As previously reported[4], CpG methylation remained relatively stable across ages, while CpH methylation in neurons showed a 2.01-fold increase in early life. (**b**) At AD enhancers, levels of CpG methylation decreased in neurons of older normal adults (no/mild AD pathology), but not in individuals with moderate or severe AD pathology (Braak stage 1-2

n=38 individuals; 3-4 n=32; 5-6 n=31; $p<0.001$ for Braak stage 1-2, robust linear regression model). Age analysis was performed on CpG sites (n=531 CpGs) in enhancers relevant to AD (n=848 enhancers showing both epigenetic and associated gene transcript differences in AD, as identified in omics integration analysis). Analysis of % CpG methylation change with age, adjusted for sex, postmortem interval, and neuronal proportion. Data generated by the bisulfite padlock probe technique with a 30× methylated read threshold.

**Supplementary Table 1**. Clinical and demographic information for investigated samples

| Braak group[ǂ] | Sex | | Age | PMI |
|---|---|---|---|---|
| | Female | Male | | |
| Braak 1-2 (n=38) | 17 | 21 | 82.55 ± 1.78 | 44.91 ± 2.85 |
| Braak 3-4 (n=32) | 16 | 16 | 84.72 ± 1.53 | 45.75 ± 3.57 |
| Braak 5-6 (n=31) | 14 | 17 | 81.87 ± 1.42 | 41.65 ± 3.42 |

[ǂ]AD diagnosis is significantly associated with Braak stage (R=0.78; $p<10^{-15}$, Pearson's correlation).

**Supplementary Table 2.** Genome-wide significant differentially methylated cytosines in AD[ǂ].

| chr | loc | strand | type | seq | logFC | t | *p*-value | *q*-value | B |
|---|---|---|---|---|---|---|---|---|---|
| chr3 | 184147761 | - | CG | CGG | -0.15 | -6.42 | 5.35E-09 | 6.46E-03 | 9.25 |
| chr2 | 3149222 | - | CHH | CAC | -0.24 | -6.18 | 1.54E-08 | 9.31E-03 | 8.39 |
| chr6 | 107988290 | - | CG | CGC | 0.13 | 6.06 | 2.64E-08 | 9.77E-03 | 7.93 |
| chr8 | 145165716 | - | CHH | CAC | -0.13 | -6.00 | 3.49E-08 | 9.77E-03 | 7.66 |
| chr21 | 38183814 | + | CG | CGA | 0.11 | 5.97 | 4.05E-08 | 9.77E-03 | 7.53 |
| chr1 | 44471534 | - | CHH | CCT | -0.24 | -5.84 | 7.18E-08 | 1.37E-02 | 7.07 |
| chr11[‡] | 117434522 | + | CHH | CAC | -0.06 | -5.82 | 7.96E-08 | 1.37E-02 | 7.03 |
| chr3 | 14584915 | + | CG | CGG | 0.16 | 5.66 | 1.59E-07 | 2.39E-02 | 6.34 |
| chr12 | 124975467 | - | CHG | CTG | -0.22 | -5.61 | 2.01E-07 | 2.70E-02 | 6.14 |
| chr7 | 111799521 | - | CHG | CAG | -2.67 | -5.50 | 3.23E-07 | 3.77E-02 | 5.85 |
| chr5 | 149652997 | - | CHH | CTA | -0.34 | -5.48 | 3.43E-07 | 3.77E-02 | 5.63 |
| chr22 | 48915491 | - | CG | CGA | 0.15 | 5.44 | 4.01E-07 | 4.04E-02 | 5.66 |
| chr5 | 139188287 | - | CHH | CAC | -0.15 | -5.38 | 5.31E-07 | 4.93E-02 | 5.45 |

[ǂ]Differentially methylated cytosine sites (individual CpGs and CpHs) corresponding to the severity of tangle pathology (Braak stage), after controlling for age, sex, postmortem interval, and neuronal subtype; $q<0.05$, robust linear regression model.

[‡]Located in enhancer at *DSCAML1* intron 3

**Supplementary Table 3.** Epigenetic disruption of genes identified by AD GWAS

| SNP id | Chromosome | Location | Annotated gene | Most significant DMR associated with gene |
|---|---|---|---|---|
| rs6656401 | 1 | 207692049 | CR1 | |
| rs6733839 | 2 | 127892810 | BIN1 | chr2:127827386-127829645; hypomethylated; $q$=1.30E-03 |
| rs35349669 | 2 | 234068476 | INPP5D | |
| rs190982 | 5 | 88223420 | MEF2C | chr5:87847668-87848509; hypomethylated; $q$=9.11E-03 |
| rs9271192 | 6 | 32578530 | HLA-DRB5–HLA-DRB1 | |
| rs10948363 | 6 | 47487762 | CD2AP | |
| rs2718058 | 7 | 37841534 | NME8 | |
| rs1476679 | 7 | 100004446 | ZCWPW1 | |
| rs11771145 | 7 | 143110762 | EPHA1 | |
| rs28834970 | 8 | 27195121 | PTK2B | |
| rs9331896 | 8 | 27467686 | CLU | |
| rs10838725 | 11 | 47557871 | CELF1 | |
| rs983392 | 11 | 59923508 | MS4A6A | |
| rs10792832 | 11 | 85867875 | PICALM | |
| rs11218343 | 11 | 121435587 | SORL1 | chr11:121443047-121443147; hypermethylated; $q$=2.98E-02 |
| rs17125944 | 14 | 53400629 | FERMT2 | |
| rs10498633 | 14 | 92926952 | SLC24A4, RIN3 | |
| rs8093731g | 18 | 29088958 | DSG2 | |
| rs4147929 | 19 | 1063443 | ABCA7 | |
| rs3865444g | 19 | 51727962 | CD33 | |
| rs7274581 | 20 | 55018260 | CASS4 | |

Red indicates AD GWAS genes that had differentially methylated enhancers in AD neurons

**Supplementary Table 4.** Differentially expressed DMRs with detectable eRNAs levels.

| chr | loc1 | loc2 | logFC | t | p-value | q-value | B |
|---|---|---|---|---|---|---|---|
| chr1 | 4762751 | 4764047 | 0.260 | 4.326 | 2.42E-04 | 2.79E-02 | 0.073 |
| chr1 | 4834309 | 4835632 | 0.186 | 3.673 | 1.24E-03 | 4.60E-02 | -0.879 |
| chr5 | 14710706 | 14711092 | 0.150 | 3.841 | 8.16E-04 | 4.10E-02 | -0.493 |
| chr6 | 35025997 | 35027668 | 0.160 | 3.860 | 7.77E-04 | 4.05E-02 | -0.458 |
| chr6 | 163986982 | 163987077 | -0.262 | -3.800 | 9.02E-04 | 4.25E-02 | -0.746 |
| chr7 | 28811076 | 28812124 | -0.314 | -3.645 | 1.33E-03 | 4.71E-02 | -1.400 |
| chr7 | 44272710 | 44273472 | 0.300 | 3.756 | 1.01E-03 | 4.39E-02 | -1.066 |
| chr7 | 157394382 | 157395000 | 0.251 | 3.775 | 9.61E-04 | 4.32E-02 | -1.073 |
| chr7 | 158253410 | 158255115 | 0.181 | 3.688 | 1.19E-03 | 4.50E-02 | -0.852 |
| chr8 | 145813688 | 145816526 | 0.207 | 3.719 | 1.10E-03 | 4.43E-02 | -0.854 |
| chr9 | 124537337 | 124538703 | 0.272 | 3.948 | 6.25E-04 | 3.75E-02 | -0.479 |
| chr9 | 132686066 | 132687101 | -0.187 | -3.936 | 6.43E-04 | 3.82E-02 | -0.284 |
| chr9 | 138676950 | 138677850 | 0.298 | 4.415 | 1.93E-04 | 2.56E-02 | 0.692 |
| chr9 | 140777461 | 140778562 | 0.190 | 3.805 | 8.91E-04 | 4.25E-02 | -0.626 |
| chr10 | 123258453 | 123261133 | -0.224 | -3.731 | 1.07E-03 | 4.40E-02 | -0.772 |
| chr11 | 13379017 | 13379596 | 0.359 | 4.439 | 1.82E-04 | 2.52E-02 | -0.047 |
| chr11 | 113978817 | 113980939 | 0.151 | 3.776 | 9.57E-04 | 4.32E-02 | -0.637 |
| chr12 | 111712396 | 111713562 | 0.363 | 4.718 | 9.02E-05 | 2.03E-02 | 0.750 |
| chr12 | 123561676 | 123563254 | 0.302 | 3.966 | 5.96E-04 | 3.72E-02 | -0.483 |
| chr12 | 131056904 | 131059213 | 0.202 | 3.613 | 1.43E-03 | 4.89E-02 | -1.055 |
| chr13 | 113625075 | 113625886 | 0.325 | 5.824 | 5.81E-06 | 1.48E-02 | 2.934 |
| chr14 | 93422062 | 93423490 | 0.215 | 3.998 | 5.51E-04 | 3.66E-02 | -0.360 |
| chr16 | 9979424 | 9979700 | 0.301 | 4.323 | 2.44E-04 | 2.79E-02 | -0.445 |
| chr17 | 1918685 | 1919074 | 0.326 | 3.635 | 1.36E-03 | 4.76E-02 | -1.369 |
| chr17 | 77075771 | 77076892 | 0.362 | 3.702 | 1.15E-03 | 4.45E-02 | -1.289 |
| chr17 | 77393769 | 77394251 | 0.333 | 3.902 | 7.00E-04 | 3.96E-02 | -0.730 |
| chr17 | 77409547 | 77411708 | 0.329 | 4.302 | 2.57E-04 | 2.82E-02 | 0.460 |
| chr17 | 77454073 | 77455189 | 0.293 | 3.752 | 1.02E-03 | 4.39E-02 | -0.729 |
| chr17 | 77461230 | 77462123 | 0.330 | 3.597 | 1.49E-03 | 4.93E-02 | -1.057 |
| chr17 | 77529375 | 77531687 | 0.284 | 4.149 | 3.77E-04 | 3.15E-02 | 0.158 |
| chr17 | 77537049 | 77541580 | 0.293 | 4.058 | 4.74E-04 | 3.42E-02 | -0.006 |
| chr18 | 70304677 | 70305411 | 0.342 | 3.940 | 6.37E-04 | 3.79E-02 | -0.313 |
| chr21 | 45748293 | 45749727 | 0.257 | 4.196 | 3.36E-04 | 3.09E-02 | 0.126 |
| chr22 | 31151672 | 31151997 | 0.361 | 3.768 | 9.76E-04 | 4.33E-02 | -1.222 |
| chr22 | 31272039 | 31275558 | 0.253 | 4.261 | 2.85E-04 | 2.90E-02 | 0.308 |
| chr22 | 48989931 | 48991490 | 0.216 | 4.134 | 3.91E-04 | 3.15E-02 | -0.044 |

**Supplementary Table 5.** Pathway analysis† for networks identified by OmicsIntegrator

| | Process networks | *p*-value | *q*-value |
|---|---|---|---|
| UBC | Inflammation_MIF signaling | 2.19E-10 | 3.41E-08 |
| | DNA damage_Checkpoint | 3.15E-09 | 2.46E-07 |
| | Cell cycle_G1-S | 5.95E-09 | 3.11E-07 |
| | Cell cycle_G0-G1 | 1.37E-08 | 5.35E-07 |
| | Inflammation_Protein C signaling | 2.40E-07 | 7.50E-06 |
| | Cell cycle_G1-S Interleukin regulation | 7.17E-07 | 1.86E-05 |
| | Immune response_BCR pathway | 2.07E-06 | 4.61E-05 |
| | Reproduction_FSH-beta signaling pathway | 5.72E-06 | 1.12E-04 |
| | Cell cycle_G1-S Growth factor regulation | 1.05E-05 | 1.82E-04 |
| | Signal Transduction_TGF-beta, GDF and Activin signaling | 1.19E-05 | 1.83E-04 |
| CUL3 | Cell cycle_G1-S Growth factor regulation | 5.66E-11 | 7.86E-09 |
| | Signal transduction_ESR1-membrane pathway | 4.15E-10 | 2.89E-08 |
| | Reproduction_Progesterone signaling | 1.35E-07 | 6.25E-06 |
| | Apoptosis_Anti-Apoptosis mediated by external signals via PI3K/AKT | 3.86E-07 | 1.34E-05 |
| | Cardiac development_FGF_ErbB signaling | 1.48E-06 | 3.65E-05 |
| | Signal transduction_Androgen receptor signaling cross-talk | 1.58E-06 | 3.65E-05 |
| | Translation_Regulation of initiation | 1.84E-06 | 3.65E-05 |
| | Reproduction_Feeding and Neurohormone signaling | 4.82E-06 | 8.38E-05 |
| | Signal transduction_WNT signaling | 5.57E-06 | 8.61E-05 |
| | Signal transduction_ESR1-nuclear pathway | 6.45E-06 | 8.97E-05 |

†Process networks, as analyzed by MetaCore

**Supplementary Table 6.** Disease enrichment† for networks identified by OmicsIntegrator

| | Disease | *p*-value | *q*-value |
|---|---|---|---|
| UBC | Lymphoma | 3.21E-23 | 5.73E-20 |
| | Pathological Conditions, Signs and Symptoms | 8.11E-22 | 7.22E-19 |
| | Virus Diseases | 5.04E-21 | 2.99E-18 |
| | Mental Disorders | 1.99E-19 | 7.42E-17 |
| | RNA Virus Infections | 2.65E-19 | 7.42E-17 |
| | Psychiatry and Psychology | 2.81E-19 | 7.42E-17 |
| | Pathologic Processes | 2.92E-19 | 7.42E-17 |
| | Chemically-Induced Disorders | 9.67E-19 | 2.15E-16 |
| | Drug-Related Side Effects and Adverse Reactions | 1.35E-18 | 2.60E-16 |
| | In-house Adverse Events | 1.46E-18 | 2.60E-16 |
| CUL3 | Amyloid Neuropathies, Familial | 1.21E-28 | 4.03E-26 |
| | Pharmacological Phenomena | 1.36E-28 | 4.03E-26 |
| | Pharmacological and Toxicological Phenomena | 1.36E-28 | 4.03E-26 |
| | Drug Resistance | 1.36E-28 | 4.03E-26 |
| | Amyloid Neuropathies | 6.44E-28 | 1.53E-25 |
| | Thyroid Carcinoma, Papillary | 6.04E-25 | 1.19E-22 |
| | Myopathy, Myofibrillar | 2.20E-23 | 3.72E-21 |
| | Nerve Sheath Neoplasms | 4.19E-23 | 6.21E-21 |
| | Aneuploidy | 5.05E-23 | 6.64E-21 |
| | Peripheral Nervous System Diseases | 1.04E-22 | 1.23E-20 |

†Disease pathways, as analyzed by MetaCore

**Supplementary Table 7.** Differentially expressed genes in the AD prefrontal cortex that were identified both in this study and the ROSMAP study by RNA-sequencing

| **Gene** | | **ROSMAP RNA-Seq** | **Study RNA-Seq**[‡] |
|---|---|---|---|
| **Gene symbol** | **Gene Location** | ***p*-value**[ǂ] | ***p*-value**[ǂ] |
| ABTB2 | chr11:34172535-34379555 | 4.15E-03 | 1.02E-04 |
| ACP1 | chr2:264140-278283 | 1.56E-03 | 7.56E-04 |
| ADAMTS2 | chr5:178537852-178772431 | 1.52E-04 | 4.64E-04 |
| ADORA2A | chr22:24813847-24838328 | 4.75E-04 | 2.51E-03 |
| AGFG2 | chr7:100136834-100165842 | 1.23E-04 | 1.08E-03 |
| AIFM3 | chr22:21319396-21335649 | 2.70E-05 | 1.85E-03 |
| AIG1 | chr6:143381633-143661441 | 4.72E-03 | 9.39E-04 |
| ALDH5A1 | chr6:24495080-24537435 | 3.17E-03 | 2.25E-03 |
| AMN1 | chr12:31824071-31882108 | 2.03E-03 | 2.83E-04 |
| AMPD2 | chr1:110158726-110174673 | 4.62E-03 | 2.45E-03 |
| AMZ2P1 | chr17:62962668-62971694 | 7.78E-04 | 1.99E-04 |
| ARHGEF10L | chr1:17866330-18024369 | 2.37E-03 | 3.91E-04 |
| ARMC10 | chr7:102715328-102740205 | 4.49E-03 | 5.19E-05 |
| ARPP19 | chr15:52839242-52862080 | 1.75E-03 | 3.82E-04 |
| ASAH1 | chr8:17913934-17942494 | 9.95E-04 | 2.93E-03 |
| ATAD1 | chr10:89511269-89601100 | 1.00E-03 | 1.31E-03 |
| BEX2 | chrX:102564274-102565974 | 2.51E-03 | 2.66E-03 |
| BTF3L4 | chr1:52521797-52556388 | 3.85E-03 | 4.28E-05 |
| CCDC88C | chr14:91737667-91884188 | 2.82E-03 | 1.53E-03 |
| CIC | chr19:42772689-42799949 | 1.23E-05 | 1.29E-03 |
| CMTM3 | chr16:66637777-66647795 | 3.80E-04 | 2.59E-04 |
| COL27A1 | chr9:116917840-117074791 | 3.45E-06 | 1.62E-06 |
| COPS8 | chr2:237993955-238009109 | 2.64E-03 | 3.75E-04 |
| COX18 | chr4:73921797-73935472 | 1.25E-03 | 2.04E-03 |
| CSNK2A1 | chr20:459116-524465 | 1.41E-03 | 2.11E-03 |
| CXXC1 | chr18:47808713-47814674 | 4.57E-03 | 2.01E-03 |
| CYP2J2 | chr1:60358980-60392462 | 1.16E-03 | 2.06E-03 |
| DAB2IP | chr9:124329336-124547809 | 1.34E-05 | 9.66E-05 |
| DCAF16 | chr4:17802278-17812381 | 2.35E-03 | 4.09E-04 |
| DGKG | chr3:185823457-186080026 | 1.76E-03 | 1.24E-04 |
| DNAJC10 | chr2:183580999-183659191 | 1.62E-04 | 8.21E-04 |
| DNAJC18 | chr5:138744279-138780180 | 1.81E-03 | 2.76E-03 |
| DYNC2LI1 | chr2:44001178-44037149 | 2.95E-03 | 1.33E-03 |
| DYNLT3 | chrX:37696010-37706890 | 1.20E-03 | 2.06E-05 |
| E2F4 | chr16:67226072-67232821 | 1.57E-03 | 9.20E-04 |
| FBXO2 | chr1:11708424-11715842 | 2.18E-06 | 2.01E-03 |
| FIZ1 | chr19:56102746-56113336 | 4.05E-03 | 2.58E-05 |
| FURIN | chr15:91411822-91426688 | 9.18E-06 | 2.47E-03 |
| GIT1 | chr17:27900487-27921072 | 1.41E-04 | 2.08E-03 |
| GK | chrX:30671476-30748725 | 2.41E-04 | 4.87E-04 |
| GNA12 | chr7:2767746-2883958 | 4.06E-03 | 1.81E-03 |
| GOLT1B | chr12:21654715-21671342 | 2.54E-03 | 4.35E-06 |
| HIRA | chr22:19318221-19435224 | 4.36E-05 | 2.75E-03 |
| HOMER3 | chr19:19040010-19052070 | 2.20E-03 | 2.46E-03 |
| HPRT1 | chrX:133594183-133654543 | 1.45E-03 | 1.09E-04 |
| HS1BP3 | chr2:20760208-20850849 | 3.78E-03 | 1.97E-03 |
| HS6ST1 | chr2:128994290-129076151 | 9.95E-04 | 5.14E-04 |
| HSD11B2 | chr16:67464555-67471456 | 1.33E-04 | 5.13E-04 |
| HSPB2 | chr11:111782966-111789574 | 2.64E-05 | 2.24E-04 |
| IFT140 | chr16:1560428-1662111 | 8.27E-04 | 4.76E-04 |

| | | | |
|---|---|---|---|
| ITFG1 | chr16:47188298-47498060 | 1.92E-03 | 1.31E-04 |
| JUND | chr19:18390563-18392432 | 1.51E-04 | 6.87E-04 |
| KATNBL1 | chr15:34432875-34502297 | 2.89E-03 | 1.53E-04 |
| KCNH3 | chr12:49932940-49952091 | 2.91E-03 | 2.13E-03 |
| KIFC3 | chr16:57792129-57896957 | 1.41E-03 | 8.76E-04 |
| LRRC4B | chr19:51020149-51071302 | 3.13E-03 | 9.00E-04 |
| LYRM7 | chr5:130506503-130541119 | 8.50E-05 | 1.26E-03 |
| MAP2K3 | chr17:21187984-21218552 | 2.75E-05 | 1.23E-03 |
| NDUFA5 | chr7:123177051-123198309 | 6.91E-04 | 5.22E-05 |
| NEU3 | chr11:74699179-74729938 | 2.42E-03 | 1.35E-03 |
| NUDT21 | chr16:56463045-56486111 | 2.15E-03 | 1.88E-05 |
| OCIAD1 | chr4:48807229-48863834 | 3.23E-03 | 1.83E-03 |
| ORAI2 | chr7:102073553-102097268 | 2.80E-03 | 1.40E-03 |
| PCCB | chr3:135969148-136056738 | 2.33E-03 | 6.74E-05 |
| PFN2 | chr3:149682691-149768575 | 1.24E-03 | 8.69E-05 |
| PLXNB2 | chr22:50713408-50746056 | 1.90E-03 | 3.02E-03 |
| PURG | chr8:30853321-30891231 | 9.07E-04 | 7.52E-04 |
| RAPGEF1 | chr9:134452157-134615461 | 2.09E-03 | 8.49E-04 |
| RBM38 | chr20:55966463-55984389 | 3.66E-04 | 2.66E-03 |
| SALL2 | chr14:21989232-22005350 | 1.91E-04 | 2.82E-03 |
| SCRIB | chr8:144873090-144897549 | 1.20E-03 | 1.24E-03 |
| SETD1A | chr16:30968615-30996437 | 3.56E-04 | 6.98E-04 |
| SLC25A40 | chr7:87462883-87505672 | 2.02E-03 | 8.38E-05 |
| SLC4A11 | chr20:3208063-3219836 | 1.88E-07 | 1.89E-03 |
| SMIM15 | chr5:60453536-60458301 | 7.88E-04 | 1.88E-03 |
| SMTN | chr22:31460091-31500743 | 3.03E-03 | 1.22E-03 |
| SPECC1L | chr22:24666786-24813708 | 1.05E-04 | 7.30E-05 |
| SRCAP | chr16:30709530-30755602 | 9.94E-05 | 2.31E-03 |
| SRRT | chr7:100472733-100486285 | 1.36E-03 | 2.79E-04 |
| SSBP2 | chr5:80708840-81047616 | 4.13E-03 | 1.67E-03 |
| TATDN2 | chr3:10289707-10322902 | 4.53E-03 | 2.08E-03 |
| TFCP2 | chr12:51487446-51566926 | 2.10E-03 | 1.23E-03 |
| TFDP2 | chr3:141663277-141868386 | 2.22E-03 | 2.32E-03 |
| TM6SF1 | chr15:83776159-83813606 | 4.36E-03 | 3.11E-03 |
| TMA16 | chr4:164415594-164441691 | 3.86E-03 | 2.73E-03 |
| TMEM14B | chr6:10747992-10852986 | 2.38E-03 | 2.60E-03 |
| TNIP1 | chr5:150409506-150473138 | 9.91E-05 | 3.43E-04 |
| TRPC1 | chr3:142442916-142526730 | 4.65E-04 | 1.07E-04 |
| UCHL5 | chr1:192981380-193029237 | 7.34E-04 | 2.98E-03 |
| USF2 | chr19:35759881-35770724 | 4.62E-03 | 2.15E-03 |
| VAV3 | chr1:108113782-108507766 | 1.19E-04 | 9.40E-04 |
| VPS29 | chr12:110928902-110939922 | 4.00E-03 | 1.31E-03 |
| VWA5B2 | chr3:183948217-183960117 | 2.89E-05 | 9.76E-04 |
| XKR8 | chr1:28285973-28294607 | 2.83E-04 | 4.69E-04 |
| ZBED1 | chrX:2404455-2419008 | 3.60E-06 | 7.00E-04 |
| ZMYND8 | chr20:45837859-45985567 | 7.78E-05 | 1.73E-04 |
| ZNF219 | chr14:21558205-21572881 | 1.19E-04 | 1.72E-03 |
| ZNF358 | chr19:7580178-7585912 | 9.96E-04 | 2.03E-03 |
| ZNF385A | chr12:54762917-54785082 | 2.20E-03 | 1.78E-03 |
| ZNF441 | chr19:11877815-11894893 | 5.58E-04 | 1.18E-03 |
| ZNF768 | chr16:30535325-30538142 | 2.82E-03 | 2.21E-03 |
| ZRANB2 | chr1:71528974-71546980 | 1.03E-03 | 6.43E-04 |

[ƚ]all differentially expressed genes are $q$<0.05, robust linear regression model. Genes are differentially expressed with AD pathology after controlling for age, sex, postmortem interval, RIN, neuronal proportion, and in ROSMAP data, years of education

[ǂ]RNA-seq in this study

**Supplementary Table 8.** Correlation between DNA methylation at cytosine in enhancers targeting *BACE1* and *BACE1* gene expression.

| chr | loc | strand | type | seq | *p*-value | coefficients | *q*-value |
|---|---|---|---|---|---|---|---|
| chr11 | 117609126 | - | CG | CGT | 4.93E-01 | 0.255 | 7.39E-01 |
| chr11 | 117609150 | - | CG | CGT | 6.33E-02 | 0.731 | 2.85E-01 |
| chr11 | 117609170 | - | CG | CGT | 2.28E-01 | 0.421 | 4.44E-01 |
| chr11 | 117609208 | - | CHH | CAT | 3.17E-02 | 0.723 | 2.28E-01 |
| chr11 | 117609223 | - | CHG | CTG | 9.97E-01 | -0.009 | 9.97E-01 |
| chr11 | 117609229 | - | CHH | CAT | 1.64E-01 | 0.479 | 4.18E-01 |
| chr11 | 117609258 | - | CHH | CAC | 2.66E-02 | 1.030 | 2.28E-01 |
| chr11 | 117609260 | - | CHH | CAC | 1.06E-01 | 0.684 | 3.94E-01 |
| chr11 | 117625155 | - | CG | CGA | 4.81E-02 | 1.005 | 2.48E-01 |
| chr11 | 117625157 | - | CHH | CAC | 4.78E-02 | 0.975 | 2.48E-01 |
| chr11 | 117625158 | - | CHH | CCA | 1.81E-01 | 16.039 | 4.18E-01 |
| chr11 | 117625159 | - | CHH | CCC | 6.15E-01 | -2.325 | 8.12E-01 |
| chr11 | 117625171 | - | CHH | CCT | 9.47E-01 | -0.161 | 9.74E-01 |
| chr11 | 117625177 | - | CHG | CTG | 6.25E-01 | -2.531 | 8.12E-01 |
| chr11 | 117625181 | - | CG | CGA | 6.86E-01 | 0.235 | 8.23E-01 |
| chr11 | 117625182 | - | CHG | CCG | 3.20E-01 | 4.216 | 5.46E-01 |
| chr11 | 117625183 | - | CHH | CCC | 6.32E-01 | 1.301 | 8.12E-01 |
| chr11 | 117625192 | - | CHG | CTG | 8.27E-01 | -0.168 | 9.05E-01 |
| chr11 | 117625196 | - | CHH | CCC | 6.59E-01 | 1.204 | 8.18E-01 |
| chr11 | 117625213 | - | CHH | CAT | 4.77E-01 | 0.323 | 7.39E-01 |
| chr11 | 117625219 | - | CHH | CTC | 1.44E-01 | -0.939 | 4.18E-01 |
| chr11[ǂ] | 117625228 | - | CHH | CAC | 3.23E-03 | -1.100 | 4.93E-02 |
| chr11 | 117625255 | - | CHH | CTC | 1.09E-01 | 0.650 | 3.94E-01 |
| chr11 | 117625259 | - | CHH | CCT | 3.19E-01 | 4.117 | 5.46E-01 |
| chr11 | 117625261 | - | CHH | CAC | 8.97E-01 | 0.052 | 9.49E-01 |
| chr11 | 117625286 | - | CHH | CCC | 5.48E-01 | 4.775 | 7.89E-01 |
| chr11 | 117642665 | + | CHG | CAG | 4.11E-03 | -15.535 | 4.93E-02 |
| chr11 | 117642675 | + | CHH | CCC | 1.73E-01 | -21.583 | 4.18E-01 |
| chr11 | 117642676 | + | CHH | CCT | 3.33E-01 | 11.427 | 5.46E-01 |
| chr11 | 117642679 | + | CHH | CCT | 1.38E-01 | 4.800 | 4.18E-01 |
| chr11 | 117642690 | + | CHG | CAG | 1.86E-01 | -3.045 | 4.18E-01 |
| chr11 | 117642720 | + | CHG | CAG | 7.27E-01 | 0.406 | 8.44E-01 |
| chr11 | 117642731 | + | CHG | CAG | 2.35E-01 | -1.537 | 4.44E-01 |
| chr11 | 117642736 | + | CHH | CTC | 2.01E-01 | 0.859 | 4.26E-01 |
| chr11 | 117642739 | + | CHG | CAG | 1.09E-04 | 2.285 | 3.91E-03 |
| chr11 | 117642784 | + | CHG | CTG | 8.30E-01 | 0.935 | 9.05E-01 |

[ǂ]enhancer site shown in Supplementary Fig. 9.

## Supplementary Methods

### Isolation of neuronal nuclei by flow cytometry

Neuronal nuclei were separated using a flow cytometry-based approach, similar to as previously described[5, 6]. Human brain tissue (250 mg) for each sample was minced in 2 mL PBSTA (0.3 M sucrose, 1X phosphate buffered saline (PBS), 0.1% Triton X-100). Samples were then homogenized in PreCellys CKMix tubes with a Minilys (Bertin Instruments) set at 3,000 rpm for three 5 sec intervals, 5 min on ice between intervals. Samples homogenates were filtered through Miracloth (EMD Millipore), followed by a rinse with an additional 2 mL of PBSTA. Samples were then place on a sucrose cushion (1.4 M sucrose) and nuclei were pelleted by centrifugation at 4,000 × g for 30 min 4°C using a swinging bucket rotor. For each sample, the supernatant was removed and the pellet was incubated in 700 µl of 1X PBS on ice for 20 min. The nuclei were then gently resuspended and blocking mix (100 µl of 1X PBS with 0.5% BSA (Thermo Fisher Scientific) and 10% normal goat serum (Gibco) was added to each sample. NeuN-488 (1:500; Abcam) was added and samples were incubated 45 min at 4°C with gentle mixing. Immediately prior to flow cytometry sorting, nuclei were stained with 7-AAD (Thermo Fisher Scientific) and passed through a 30 µM filter (SystemX). Nuclei positive for 7-AAD and either NeuN+ (neuronal) or NeuN– (non-neuronal) were sorted using a MoFlo Astrios (Beckman Coulter) running Summit 6.3 by the Van Andel Research Institute Flow Cytometry Core. Approximately 1 million NeuN+ nuclei were sorted for each sample. NeuN+ sample purity (~96%) was confirmed by reanalysis on the sorter, and was confirmed by RNA analysis showing an enrichment of neuronal markers in the NeuN+ (but not NeuN–) fraction (Supplementary Fig. 1). Immediately, after sorting nuclei were placed on ice and then precipitated with 0.3 M sucrose, 4.2 mM $CaCl_2$ and 2.5 mM $Mg(Ace)_2$ and centrifugation at 1,786 × g for 15 min at 4°C. The

supernatant was removed from NeuN+ and NeuN– samples and pellets were stored at -80°C. Genomic DNA from each sample's NeuN+ fraction was isolated using standard phenol-chloroform extraction methods.

**DNA methylation analysis comparison**

We compared our findings with existing packages for DNA methylation analysis. We used DMRseq[7] to detect genomic regions with DNA methylation changes associated with increasing Braak stage pathology (parameters: % methylation cutoff=0.001; cytosine minimum per region=3), adjusting for age, sex, postmortem interval, and neuronal subtype proportion. We found a strong correlation between the results of DMRseq and our analysis ($p<10^{-15}$, Pearson's correlation).

**Enhancer DNA methylation association with RNA levels**

We examined our DNA methylation and corresponding RNA-seq data (n=25 individuals) to determine whether there was a relationship between DNA methylation changes with AD pathology and mRNA level changes with AD pathology. First, we determined the average DNA methylation at each enhancer region. Enhancers were matched to their respective target genes (determined from the chromatin conformation analysis described above). We then determined the Pearson's correlation between residual DNA methylation status change with AD Braak stage (adjusted for age, sex, postmortem interval, and neuronal subtype) and residual mRNA level change with AD Braak stage (adjusted for sex, age, postmortem interval, neuronal proportion, and RIN). DNA methylation and target gene expression changes with AD pathology were

evaluated for enhancers previously identified to be differentially methylated in AD (significant DMR) or enhancers unchanged in AD (non-significant DMR).

eRNAs are non-coding transcripts expressed from enhancers, whose expression has been previously shown to correlate with enhancer activation and mRNA expression of the target gene[8]. Our RNA-seq data (n=25 individuals) was also used to profile eRNA expression from enhancer regions identified in our previous DNA methylation analysis. These enhancer regions were combined with the Gencode v19 primary assembly annotation to create a new transcriptome annotation file. STAR[9] (v2.3.5a) index was generated using Ensemble GRCh37 p13 primary assembly genome and this new transcriptome annotation. Read alignment, counting and trimmed mean of M-values normalization was performed as previously stated[10]. Lowly expressed genes and eRNAs were filtered (FPKM < 0.01) as previously published[10]. We identified eRNA expression at 2,563 brain enhancers. A robust linear model examining AD Braak stage and adjusting for age, sex, postmortem interval, neuronal cell composition, and RIN with empirical Bayes, was performed to identify differentially expressed eRNAs and genes, followed by Benjamini-Hochberg multiple testing correction (statistical significance $q<0.05$). Hypergeometric testing was used to determine whether differentially expressed eRNAs in AD were enriched at enhancer regions with differential methylation in AD.

We examined in our DNA methylation and RNA-seq data (n=25 individuals) the relationship between methylation at *DSCAML1* enhancers and *BACE1* mRNA expression. For this, we compared DNA methylation in AD-relevant *DSCAML1* enhancers found to target *BACE1* in the Hi-C analysis to the expression of the *BACE1* gene using a robust linear regression by limma and

adjusting for sex, age, postmortem interval, neuronal proportion, and RIN (Supplementary Table 8).

**Validation in independent datasets**

We used data from the Religious Orders Study (ROS) and The Memory and Aging Project (MAP)[11] (Synapse ID: syn3219045 [https://www.synapse.org/#!Synapse:syn3219045]). All individuals used in this analysis had a clinical diagnosis of unaffected/control or AD.

RNA-seq data (Synapse ID: syn3388564 [https://www.synapse.org/#!Synapse:syn3388564]) was obtained for 338 control and AD prefrontal cortex samples. RNA isolation, sequencing, and data preprocessing (including quantile normalization and Combat[12] to remove batch effects) was performed by the ROSMAP as described in detail in refs[13, 14]. Low expressed genes were removed (FPKM < 0.5 in more than 30% of samples). We then applied a robust linear regression using limma[15] with Braak stage as the independent variable, log2 transformed FPKM values as the dependent variable, and age, sex, postmortem interval, neuronal cell proportion, and RIN (verified by TIN) as covariates. Differentially expressed genes were selected after Benjamini-Hochberg correction for multiple testing ($q<0.05$).

DNA methylation array (Synapse ID: syn3157275 [https://www.synapse.org/#!Synapse:syn3157275]) data generated by Illumina HumanMethylation450 BeadChip was used to examine the effects of *DSCAML1* intron 3 methylation on *BACE1* expression (177 AD samples with DNA methylation array and RNA-seq, of which 101 were Braak stage≤4) and AD pathology and symptoms (465 control and AD

samples with DNA methylation array and pathological/cognitive testing). Detailed information on DNA methylation data collection from the human prefrontal cortex for the ROSMAP study is provided in ref[16]. DNA methylation array batch effects were removed by Combat[12]. First, we examined the correlation between DNA methylation at cg07533617 (or averaged *DSCAML1* intron 3 methylation) and *BACE1* gene expression, after adjusting for age, sex, postmortem interval, years of education, neuronal cell proportion, and RIN, using a robust linear regression by limma[15, 17]. Next, we determined the relationship between DNA methylation status at *DSCAML1* intron 3 and AD pathology. Uniform assessment of AD pathology, including β-amyloid and tangles, had been conducted for ROSMAP samples as previously described[18, 19]. We used robust linear regressions by limma[15, 17], adjusting for age, sex, postmortem interval, years of education, and neuronal cell proportion, to determine the association between DNA methylation at cg07533617 (or averaged *DSCAML1* intron 3 methylation) and pathological variables: amyloid level and neurofibrillary tangle density. Finally, we determined whether DNA methylation at *DSCAML1* intron 3 was linked to the progression of cognitive decline. Participants in the ROSMAP study had undergone comprehensive annual cognitive assessments for many years, as previously described[20]. Annual composite scores for global cognitive function, as well as summary measures for episodic memory (7 tests), semantic memory (3 tests), working memory (3 tests), processing speed (2 tests), and visuospatial ability (2 tests) were used (total of 6 cognitive domains)[20]. We used a linear mixed model to examine the relationship between DNA methylation at cg07533617 (or averaged *DSCAML1* intron 3 methylation) and the rate of decline in each cognitive domain. The model used annual cognitive scores as continuous longitudinal outcomes and included the factors of DNA methylation level, time in years prior to death, DNA methylation by time interaction, as well as a random intercept

for each person. The model was adjusted for age, sex, years of education, postmortem interval, and neuronal cell proportion. The models employed in this study are analogous to those used in other studies involving pathological/clinical ROSMAP data[15, 17].

To further examine the influence of enhancers within *DSCAML1* intron 3 on *BACE1* expression, we performed an analysis examining the relationship between cis-acting haplotypes and *BACE1* transcript levels in controls and AD patients. For this analysis, we used imputed genotype data that was obtained from the ROSMAP study (Synapse ID: syn3157329 [https://www.synapse.org/#!Synapse:syn3157329]). There were 278 AD patients and controls with both genotype and RNA-seq data in the ROSMAP. As described[21], only individuals of European ancestry were included in this analysis to minimize population heterogeneity. The sample-level quality control criteria excluded samples that had a genotype success rate below 95%, a discordance between inferred and reported sex, and excess inter/intraheterozygosity. The SNP-level quality control criteria excluded SNPs with Hardy-Weighberg equilibrium ($p$<0.001), MAF < 0.01, genotype call rate < 0.95, mishap test < $1x10^{-9}$. Population outliers were removed using EIGENSTRAT[22] with default parameters. We performed a cis-haplotype analysis on the extended *BACE1* genomic area (± 500 kb centered on *BACE1* and including the *DSCAML1* gene); this analysis involved 5,533 SNPs from which there were 53 haplotypes identified by Haploview[23] (with 2 or more SNPs per haplotype). Gene expression values for all genes were normalized for eQTL analysis using the following procedure: 1) Low expressed genes were removed (FPKM < 0.5 in more than 30% of samples); 2) The distribution of FPKMs in each sample was quantile normalized to the average empirical distribution observed across all samples; 3) Log2 transformed FPKM values were used. We performed a linear regression to

assess the effect of genotype on *BACE1* expression, with sex, age, postmortem interval, Braak stage, years of education, neuronal cell proportion, and RIN as covariates. Then we generated a combined p value for SNPs within each haplotype using the Fisher's method[24]. Benjamini-Hochberg method was used for false discovery rate control. This analysis was repeated to examine whether cis-acting genetic variation in *DSCAML1* intron 3 affected the transcript levels of nearby cis-genes: *BACE1*, *CEP164*, and *DSCAML1* (chr11:117,000,000-117,800,000, genomic area centered on *DSCAML1* intron 3). This analysis involved 4,305 SNPs from which 34 haplotypes were determined by Haploview[23] (with 2 or more SNPs per haplotype). Benjamini-Hochberg adjustment for multiple testing was applied to the combined p values for the 34 haplotypes for each gene.

## Supplementary References


1. Luo, C., *et al.* Single-cell methylomes identify neuronal subtypes and regulatory elements in mammalian cortex. *Science* **357**, 600-604 (2017).
2. Drummond, E.S., Nayak, S., Ueberheide, B. & Wisniewski, T. Proteomic analysis of neurons microdissected from formalin-fixed, paraffin-embedded Alzheimer's disease brain tissue. *Scientific reports* **5**, 15456 (2015).
3. Tuncbag, N., *et al.* Network-Based Interpretation of Diverse High-Throughput Datasets through the Omics Integrator Software Package. *PLoS computational biology* **12**, e1004879 (2016).
4. Lister, R., *et al.* Global epigenomic reconfiguration during mammalian brain development. *Science* **341**, 1237905 (2013).
5. Yu, P., McKinney, E.C., Kandasamy, M.M., Albert, A.L. & Meagher, R.B. Characterization of brain cell nuclei with decondensed chromatin. *Developmental neurobiology* **75**, 738-756 (2015).
6. Matevossian, A. & Akbarian, S. Neuronal nuclei isolation from human postmortem brain tissue. *Journal of visualized experiments : JoVE* (2008).
7. Korthauer, K., Chakraborty, S., Benjamini, Y. & Irizarry, R.A. Detection and accurate false discovery rate control of differentially methylated regions from whole genome bisulfite sequencing. *Biostatistics* (2018).
8. Arner, E., *et al.* Transcribed enhancers lead waves of coordinated transcription in transitioning mammalian cells. *Science* **347**, 1010-1014 (2015).
9. Dobin, A., *et al.* STAR: ultrafast universal RNA-seq aligner. *Bioinformatics* **29**, 15-21 (2013).
10. Dong, X., *et al.* Enhancers active in dopamine neurons are a primary link between genetic variation and neuropsychiatric disease. *Nature neuroscience* **21**, 1482-1492 (2018).
11. Bennett DA, B.A., Boyle PA, Barnes LL, Wilson RS, Schneider JA. Religious Orders Study and Rush Memory and Aging Project
*Journal of Alzheimer's Disease* (in press).
12. Johnson, W.E., Li, C. & Rabinovic, A. Adjusting batch effects in microarray expression data using empirical Bayes methods. *Biostatistics* **8**, 118-127 (2007).
13. White, C.C., *et al.* Identification of genes associated with dissociation of cognitive performance and neuropathological burden: Multistep analysis of genetic, epigenetic, and transcriptional data. *PLoS medicine* **14**, e1002287 (2017).
14. Bennett, D.A., Yu, L. & De Jager, P.L. Building a pipeline to discover and validate novel therapeutic targets and lead compounds for Alzheimer's disease. *Biochemical pharmacology* **88**, 617-630 (2014).
15. Ritchie, M.E., *et al.* limma powers differential expression analyses for RNA-sequencing and microarray studies. *Nucleic Acids Res* **43**, e47 (2015).
16. De Jager, P.L., *et al.* Alzheimer's disease: early alterations in brain DNA methylation at ANK1, BIN1, RHBDF2 and other loci. *Nature neuroscience* **17**, 1156-1163 (2014).
17. Do Carmo, S., *et al.* Rescue of Early bace-1 and Global DNA Demethylation by S-Adenosylmethionine Reduces Amyloid Pathology and Improves Cognition in an Alzheimer's Model. *Scientific reports* **6**, 34051 (2016).

18. Boyle, P.A., *et al.* Much of late life cognitive decline is not due to common neurodegenerative pathologies. *Ann Neurol* **74**, 478-489 (2013).
19. Bennett, D.A., Schneider, J.A., Wilson, R.S., Bienias, J.L. & Arnold, S.E. Education modifies the association of amyloid but not tangles with cognitive function. *Neurology* **65**, 953-955 (2005).
20. Wilson, R.S., *et al.* Conscientiousness, dementia related pathology, and trajectories of cognitive aging. *Psychology and aging* **30**, 74-82 (2015).
21. De Jager, P.L., *et al.* A genome-wide scan for common variants affecting the rate of age-related cognitive decline. *Neurobiol Aging* **33**, 1017 e1011-1015 (2012).
22. Price, A.L., *et al.* Principal components analysis corrects for stratification in genome-wide association studies. *Nat Genet* **38**, 904-909 (2006).
23. Barrett, J.C., Fry, B., Maller, J. & Daly, M.J. Haploview: analysis and visualization of LD and haplotype maps. *Bioinformatics* **21**, 263-265 (2005).
24. Dewey, M. metap: Meta-Analysis of Significance Values. in *The metap package* (https://cran.r-project.org/web/packages/metap/index.html, 2018).
