# Supplementary material for: Epigenetic dysregulation of enhancers in neurons is associated with Alzheimer’s disease pathology and cognitive symptoms
Source: Nat Commun. 2019 May 21;10:2246. doi: 10.1038/s41467-019-10101-7 (PMC6529540; doi:10.1038/s41467-019-10101-7)
Supplement: Supplementary file 2 — Description of Additional Supplementary Files [file 41467_2019_10101_MOESM2_ESM.pdf]

Description of Additional Supplementary Files

File Name: Supplementary Data 1
Description: Differentially methylated regions

File Name: Supplementary Data 2
Description: Hi-C genes interacting with differentially methylated regions

File Name: Supplementary Data 3
Description: Differentially expressed genes by RNA-seq

File Name: Supplementary Data 4
Description: Haplotype analysis

File Name: Supplementary Data 5
Description: Primers
