# Supplementary material for: Epigenetic dysregulation of enhancers in neurons is associated with Alzheimer’s disease pathology and cognitive symptoms
Source: Nat Commun. 2019 May 21;10:2246. doi: 10.1038/s41467-019-10101-7 (PMC6529540; doi:10.1038/s41467-019-10101-7)
Supplement: Supplementary file 8 — Reporting Summary [file 41467_2019_10101_MOESM8_ESM.pdf]

nature research

Corresponding author(s): NCOMMS-18-26713C

Last updated by author(s): Apr 8, 2019

Reporting Summary

Nature Research wishes to improve the reproducibility of the work that we publish. This form provides structure for consistency and transparency in reporting. For further information on Nature Research policies, see Authors & Referees and the Editorial Policy Checklist.

Statistics

For all statistical analyses, confirm that the following items are present in the figure legend, table legend, main text, or Methods section.

n/a	Confirmed	
☐	☒	The exact sample size (*n*) for each experimental group/condition, given as a discrete number and unit of measurement
☐	☒	A statement on whether measurements were taken from distinct samples or whether the same sample was measured repeatedly
☐	☒	The statistical test(s) used AND whether they are one- or two-sided<br>*Only common tests should be described solely by name; describe more complex techniques in the Methods section.*
☐	☒	A description of all covariates tested
☐	☒	A description of any assumptions or corrections, such as tests of normality and adjustment for multiple comparisons
☐	☒	A full description of the statistical parameters including central tendency (e.g. means) or other basic estimates (e.g. regression coefficient) AND variation (e.g. standard deviation) or associated estimates of uncertainty (e.g. confidence intervals)
☐	☒	For null hypothesis testing, the test statistic (e.g. *F*, *t*, *r*) with confidence intervals, effect sizes, degrees of freedom and *P* value noted<br>*Give P values as exact values whenever suitable.*
☒	☐	For Bayesian analysis, information on the choice of priors and Markov chain Monte Carlo settings
☒	☐	For hierarchical and complex designs, identification of the appropriate level for tests and full reporting of outcomes
☐	☒	Estimates of effect sizes (e.g. Cohen's *d*, Pearson's *r*), indicating how they were calculated

Our web collection on statistics for biologists contains articles on many of the points above.

Software and code

Policy information about availability of computer code

Data collection	EpiCompare: Predict tissue/cell type-specific enhancers/promoters<br>ChromHMM(v1.14): Predict chromatin state data<br>ppDesigner(v1.1): Generate padlock probes
Data analysis	Bismark(v0.17.0): Call methylation<br>Trimmomatic(v0.32): Remove adapter sequences for methylation raw data<br>R(v3.4.1): all basic data analysis<br>lumi package(v2.30.0): Transformed DNA methylation B-values to M-values<br>limma package(v3.30.13): Run a multivariate robust linear regression model with empirical Bayes<br>BACON(v1.6.0): Controll bias and inflation in association studies using the empirical null distribution<br>DMRseq(v1.2.5): detect DMR<br>CETS(v3.03): Quantify neuronal subtype proportion (glutamate and GABA)<br>CIBERSORT: Perform cell-type deconvolution based on methylation and gene expression data<br>oPOSSUM(v3.0): Perform TF analysis<br>Trim Galore(v0.11.5): Adapter trimming for neuron Hi-C data and RNA-seq raw data<br>HICCUP(v0.5.9): Map and perform quality control<br>GOTHiC(1.14.0): Identify significant interactions<br>GREAT(v3.0.0): Identify nearby genes<br>MetaCore: Pathway enrichment analysis<br>FunciSNP(v1.18.0): Find SNPs in linkage disequilibrium<br>plink(v3): calculate LD blocks<br>LDSC: obtain the computed LD Scores<br>STAR(v2.3.5a): Perform read alignment<br>RSEM(v1.2.31): Gene counting<br>EdgeR(v3.16.5): Gene expression normalization

variancePartition package(v1.12.3):quantify variation of each factor in RNA-Seq data
TIN: measure RIN
stats package(v3.3.3): RNA-Seq data multiple testing correction
OmicsIntegrator(Garnet and Forest): Identify underlying molecular pathways by merging epigenetic and transcriptomic sequencing data
Cytoscape(v3.6.0): Network visualization
Combat(v3.26.0): Remove batch effects in ROS-MAP data
lmerTest(v3.0.0): Perform linear mixed-effects model
metap package(v1.1): calculate combined-pvalue
Haploview: identify haplotypes
glmnet(v2.0.16): Perform elastic net regression in DNA methylation age calculator

Customized codes: Available at https://github.com/lipeipei0611/AD_Enh
AD_QC_Pipeline: Pre-processing DNA methylation data
DEG: Create DEG
DMR: Update DMR
FindBackgroundDMR: Rename FindBin to FindBackgroundDMR
GeneAnnotation: Create GeneAnnotation
ROSMAP_DEG: Create ROSMAP_DEG
ROSMAP_RNASeq_Methylation_Correlation: Create ROSMAP_RNASeq_Methylation_Correlation
eQTL: Create eQTL

For manuscripts utilizing custom algorithms or software that are central to the research but not yet described in published literature, software must be made available to editors/reviewers. We strongly encourage code deposition in a community repository (e.g. GitHub). See the Nature Research guidelines for submitting code & software for further information.

Data

Policy information about availability of data

All manuscripts must include a data availability statement. This statement should provide the following information, where applicable:
- Accession codes, unique identifiers, or web links for publicly available datasets
- A list of figures that have associated raw data
- A description of any restrictions on data availability

All sequencing data generated in this study are available from the NCBI Gene Expression Omnibus (GEO) database under the accession number GSE110732

Field-specific reporting

Please select the one below that is the best fit for your research. If you are not sure, read the appropriate sections before making your selection.

☒ Life sciences ☐ Behavioural & social sciences ☐ Ecological, evolutionary & environmental sciences

For a reference copy of the document with all sections, see nature.com/documents/nr-reporting-summary-flat.pdf

Life sciences study design

All studies must disclose on these points even when the disclosure is negative.

Sample size	DNA methylation status was interrogated at every cytosine site (CpG and CpH) covered by padlock probes targeting 35,288 regulatory regions across the genome of 131 samples (n=106 unique samples, 2 whole-genome amplified (WGA) control samples and 23 replicate samples).<br>RNA-sequencing to profile the mRNA transcriptome in the prefrontal cortex of 25 individuals (samples also in DNA methylation study above).
Data exclusions	In the DNA methylation analysis: There were 7 samples (5 unique samples and two replicates) that were excluded from further analyses due to poor inter-sample correlations (>10% difference).<br>No samples were excluded from the RNA-seq analysis.
Replication	For our bisulfite padlock probe (DNA methylation) library preparation: Technical and sequencing replicates confirmed a high reproducibility in sample-level methylation correlation analysis (average R for technical replicates: 0.976; average R for sequencing replicates: 0.976).<br><br>We replicated our study's results/findings with two independent datasets: 1) ROSMAP data (RNA-seq, DNA methylation arrays, SNP arrays) performed in prefrontal cortex of control and AD patients and 2) proteomics dataset in laser captured cortical neurons from controls and AD patients.
Randomization	All samples were randomized in the study (both in the isolation of neuronal nuclei and DNA methylation and RNA-seq library preparation).
Blinding	An experimenter blind to the sample key performed the isolation of neuronal nuclei and the DNA methylation and RNA-seq library preparation.

Reporting for specific materials, systems and methods

We require information from authors about some types of materials, experimental systems and methods used in many studies. Here, indicate whether each material, system or method listed is relevant to your study. If you are not sure if a list item applies to your research, read the appropriate section before selecting a response.

Materials & experimental systems

n/a	Involved in the study
☐	☒ Antibodies
☒	☐ Eukaryotic cell lines
☒	☐ Palaeontology
☒	☐ Animals and other organisms
☒	☐ Human research participants
☒	☐ Clinical data

Methods

n/a	Involved in the study
☒	☐ ChIP-seq
☐	☒ Flow cytometry
☒	☐ MRI-based neuroimaging

Antibodies

Antibodies used	NeuN Alexa Fluro 488, Abcam, product number: ab190195, concentration used: 1:500, host species: Rabbit, monoclonal Lot: gr26228-1
Validation	This antibody has been validated for flow cytometry 1:500 concentration Website states: Tested applications Suitable for: ICC/IF, Flow Cyt, IHC-Fr (http://www.abcam.com/neun-antibody-epr12763-neuronal-marker-alexa-fluor-488-ab190195.html)

Flow Cytometry

Plots

Confirm that:

☒ The axis labels state the marker and fluorochrome used (e.g. CD4-FITC).

☒ The axis scales are clearly visible. Include numbers along axes only for bottom left plot of group (a 'group' is an analysis of identical markers).

☒ All plots are contour plots with outliers or pseudocolor plots.

☒ A numerical value for number of cells or percentage (with statistics) is provided.

Methodology

Sample preparation	Human brain tissue (250 mg) for each sample was minced in 2 mL PBSTA (0.3 M sucrose, 1X phosphate buffered saline (PBS), 0.1% Triton X-100). Samples were then homogenized in PreCellys CKMix tubes with a Minilys (Bertin Instruments) set at 3,000 rpm for three 5 sec intervals, 5 min on ice between intervals. Samples homogenates were filtered through Miracloth (EMD Millipore), followed by a rinse with an additional 2 mL of PBSTA. Samples were then place on a sucrose cushion (1.4 M sucrose) and nuclei were pelleted by centrifugation at 4,000 × g for 30 min 4°C using a swinging bucket rotor. For each sample, the supernatant was removed and the pellet was incubated in 700 µl of 1X PBS on ice for 20 min. The nuclei were then gently resuspended and blocking mix (100 µl of 1X PBS with 0.5% BSA (Thermo Fisher Scientific) and 10% normal goat serum (Gibco)) was added to each sample. NeuN-488 (1:500; Abcam) was added and samples were incubated 45 min at 4°C with gentle mixing.
Instrument	MoFlo Astrios (Beckman Coulter)
Software	Summit 6.3
Cell population abundance	Approximately 1 million NeuN+ nuclei were sorted for each sample. NeuN+ sample purity (~96%) was confirmed by reanalysis on the sorter, and was confirmed by RNA analysis showing an enrichment of neuronal markers in the NeuN+ (but not NeuN–) fraction (Supplementary Fig. 1). Immediately, after sorting nuclei were placed on ice and then precipitated with 0.3 M sucrose, 4.2 mM CaCl2 and 2.5 mM Mg(Ace)2 and centrifugation at 1,786 × g for 15 min at 4°C. The supernatant was removed from NeuN+ and NeuN– samples and pellets were stored at -80°C. Genomic DNA from each sample's NeuN+ fraction was isolated using standard phenol-chloroform extraction methods
Gating strategy	Nuclei positive for 7-AAD and either NeuN+ (neuronal) or NeuN– (non-neuronal) were sorted using a MoFlo Astrios (Beckman Coulter) running Summit 6.3 by the Van Andel Research Institute Flow Cytometry Core. Gating was based on unstained, NeuN+ only, and 7-AAD only controls (each independently run to determine the gating with FSC). Approximately 1 million NeuN+ nuclei were sorted for each sample. NeuN+ sample purity (~96%) was confirmed by reanalysis on the sorter, and was confirmed by RNA analysis showing an enrichment of neuronal markers in the NeuN+ (but not NeuN–) fraction (Supplementary Fig. 1).

☒ Tick this box to confirm that a figure exemplifying the gating strategy is provided in the Supplementary Information.
